# *Txnip* deficiency causes a susceptibility to acute cold stress with brown fat dysfunction in mice

**DOI:** 10.1016/j.jbc.2025.108293

**Published:** 2025-02-11

**Authors:** Meng Zou, Katsuya Tanabe, Kikuko Amo-Shiinoki, Daisuke Kohno, Syota Kagawa, Hideki Shirasawa, Kenji Ikeda, Akihiko Taguchi, Yasuharu Ohta, Shigeru Okuya, Tetsuya Yamada, Tadahiro Kitamura, Hiroshi Masutani, Yukio Tanizawa

**Affiliations:** 1 Division of Endocrinology, Metabolism, Hematological Sciences and Therapeutics, Yamaguchi University Graduate School of Medicine, Ube, Yamaguchi, Japan; 2 Metabolic Signal Research Center, Institute for Molecular and Cellular Regulation, Gunma University, Maebashi, Gunma, Japan; 3 Department of Natural Products Chemistry, Daiichi University of Pharmacy, Fukuoka, Japan; 4 Department of Molecular Endocrinology and Metabolism, Tokyo Medical and Dental University, Tokyo, Japan; 5 Health Administration Center, Organization for University Education, Yamaguchi University, Yamaguchi, Japan; 6 Department of Clinical Laboratory Sciences, Faculty of Health Care, Tenri University, Tenri, Nara, Japan

**Keywords:** thioredoxin-interacting protein, brown adipose tissue, thermogenesis, cold stress, metabolism

## Abstract

Mammals adaptively regulate energy metabolism in response to environmental changes such as starvation and cold circumstances. Thioredoxin-interacting protein (Txnip), known as a redox regulator, serves as a nutrient sensor regulating energy homeostasis. Txnip is essential for mice to adapt to starvation, but its role in adapting to cold circumstances remains unclear. Here, we identified Txnip as a pivotal factor for maintaining nonshivering thermogenesis in mice. Txnip protein levels in brown adipose tissue (BAT) were upregulated by the acute cold exposure. *Txnip*-deficient (*Txnip*^−/−^) mice acclimated to thermoneutrality (30 °C) exhibited significant BAT enlargement and triglyceride accumulation with downregulation of BAT signature and metabolic gene expression. Upon acute cold exposure (5 °C), *Txnip*^−/−^ mice showed a rapid decline in BAT surface temperatures with the failure of increasing metabolic respiration, developing lethal hypothermia. The BAT dysfunction and cold susceptibility in *Txnip*^−/−^ mice were corrected by acclimation to 16 °C, protecting the mice from life-threatening hypothermia. Transcriptomic and metabolomic analysis using dissected BAT revealed that despite preserving glycolysis, the BAT of *Txnip*^−/−^ mice failed to activate the catabolism of branched-chain amino acids and fatty acids in response to acute cold stress. These findings illustrate that Txnip is required for maintaining basal BAT function and ensuring cold-induced thermogenesis.

Mammals possess sophisticated physiological mechanisms to adapt to challenging environmental conditions, such as prolonged starvation, endurance exercise, and cold circumstances. Regulation of nutrient metabolism to maintain energy homeostasis is essential for these adaptive responses.

Thioredoxin-interacting protein (Txnip) serves as a nutrient sensor regulating energy homeostasis across various systems (1, 2, 3, 4, 5, 6, 7, 8, 9). Txnip binds to and inhibits thioredoxin, thereby modulating the cellular redox state and promoting oxidative stress (10, 11, 12, 13). Additionally, Txnip engages in various redox-independent cellular functions, primarily relying on its role as an ancestral α-arrestin family scaffold (14, 15, 16, 17). Previous studies have shown that Txnip is a critical regulator in glucose and lipid metabolism (4, 8, 18). Furthermore, the effects of loss of Txnip have been demonstrated in *Txnip*-deficient mice, exhibiting intolerance to starvation, endurance exercise, and inflammation (5, 7, 15). These lines of evidence suggest that in mice, Txnip plays a role in adapting to physiological stresses through the regulation of energy metabolism. However, its role in nonshivering thermogenesis, a crucial aspect of energy expenditure in response to cold exposure, remains poorly understood.

Nonshivering thermogenesis serves as a fundamental mechanism for maintaining core body temperature and metabolic balance under cold stress, which is primarily mediated by brown adipose tissue (BAT) and beige adipocytes within white adipose tissue (WAT) (19). Beige adipocytes arise postnatally in response to external stimuli, such as chronic cold exposure, whereas brown adipocytes, once developed, maintain a relatively high thermogenic gene expression, remaining primed for thermogenesis, even without stimulation (20). In response to an adaptive challenge, BAT efficiently oxidizes glucose, lipids, and amino acids to rapidly generate heat through uncoupling protein 1 (Ucp1)-mediated proton gradient uncoupling of mitochondrial respiration (19, 21). Despite significant research on the various signaling pathways and metabolic processes of nonshivering thermogenesis (22, 23, 24, 25, 26), the precise role of Txnip in this context remains unclear. Hence, this study aimed to explore the effects of *Txnip* deficiency on nonshivering thermogenesis under various temperature conditions. Using *Txnip*-deficient mice and their BAT, we found that Txnip plays a pivotal role in preserving BAT thermogenesis and oxidative metabolism and is essential for adaptive thermogenesis in response to sudden cold stress.

## Results

### *Txnip* deficiency causes lethal hypothermia under acute cold stress with a lack of BAT thermogenic response

**Figure 1:**
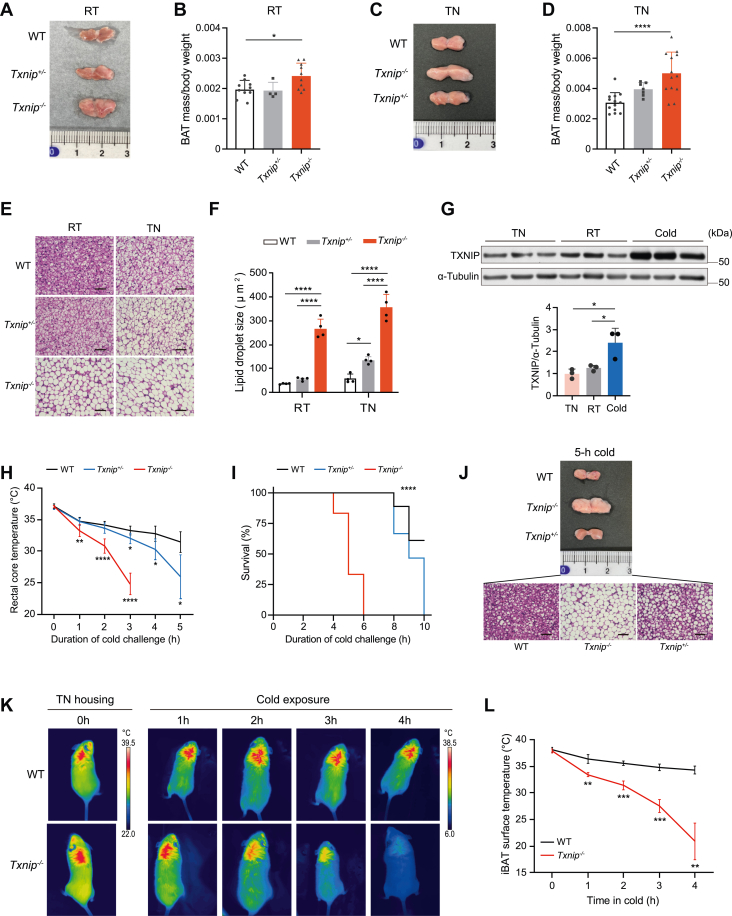
***Txnip*-deficient mice exhibited altered BAT morphology and severe intolerance to acute cold exposure.***A*, representative image of interscapular BAT from 10-week-old WT, *Txnip*-haploinsufficient (*Txnip*^*+/−*^), and *Txnip*-deficient (*Txnip*^*−/−*^) mice housed at room temperature (RT, 23 °C). *B*, interscapular BAT mass normalized by body weight in 10-week-old WT, *Txnip*^*+/−*^, and *Txnip*^*−/−*^ mice housed at RT on a chow diet (n = 10,4,10). *C*, representative image of interscapular BAT from 10-week-old WT, *Txnip*^*−/−*^, and *Txnip*^*+/−*^ mice acclimated to thermoneutral (TN, 30 °C) conditions for 1 week. *D*, interscapular BAT mass normalized by body weight in 10-week-old WT, *Txnip*^*+/−*^, and *Txnip*^*−/−*^ mice acclimated to TN conditions on a chow diet (n = 13,7,13). *E*, H&E staining of interscapular BAT from 10-week-old WT, *Txnip*^*+/−*^, and *Txnip*^*−/−*^ mice housed at RT and TN conditions. The scale bar represents 50 μm. *F*, quantification of lipid droplet size from BAT H&E-stained slides (n = 4 per group). *G*, Western blot and TXNIP quantification in BAT from WT mice housed at TN, RT, and after 6 h cold exposure at 5 °C (from RT). α-Tubulin was used as a loading control (n = 3 per group). *H*, rectal core temperatures of WT, *Txnip*^*+/−*^, and *Txnip*^*−/−*^ mice exposed to acute cold at 5 °C after TN acclimation for 1 week (n = 10, 8, 10). *I*, survival rates of WT, *Txnip*^*+/−*^, and *Txnip*^*−/−*^ mice exposed to acute cold at 5 °C after TN acclimation for 1 week (n = 18, 15, 18). *J*, representative interscapular BAT image and H&E staining from WT, *Txnip*^*−/−*^, and *Txnip*^*+/−*^ mice exposed to acute cold for 5 h after TN acclimation for 1 week. The scale bar represents 50 μm. *K*, representative infrared images of WT and *Txnip*^*−/−*^ mice prehoused in TN, followed by acute cold exposure. *L*, interscapular BAT surface temperatures of WT and *Txnip*^*−/−*^ mice during acute cold exposure after TN acclimation (n = 4 per group). Data: mean ± SD. Statistical analyses: one-way ANOVA with Bonferroni's *post hoc* test (*B*, *D*, *F*, and *G*), two-way ANOVA with Bonferroni's *post hoc* test (*H*, and *L*), and log-rank (Mantel–Cox) test (*I*). ∗*p* < 0.05, ∗∗*p* < 0.01, ∗∗∗*p* < 0.001, and ∗∗∗∗*p* < 0.0001. See also Figs. S1–S3. BAT, brown adipose tissue; Txnip, thioredoxin-interacting protein.

As previously described (8), *Txnip*^−/−^ mice exhibited an increase in subcutaneous WAT mass (Fig. S1, *A*–*D*), while maintaining a total body weight similar to that of WT mice (Fig. S1, *E* and *F*). To investigate the effect of Txnip deficiency on BAT physiology, we initially examined BAT morphology in *Txnip*^−/−^ and *Txnip*-haploinsufficient (*Txnip*^+/−^) mice. Txnip expression in BAT declined in accordance with the loss of gene copy number (Fig. S1*G*). The BAT from *Txnip*^−/−^ mice appeared paler and larger than other genotypes (Fig. 1, *A* and *B*), particularly evident under thermoneutral (TN, 30 °C) conditions (Fig. 1, *C* and *D*). Microscopically, brown adipocytes of *Txnip*^−/−^ mice exhibited unilocular hypertrophic lipid droplets, a characteristic that intensified upon TN acclimation (Fig. 1, *E* and *F*). *Txnip*^+/−^ mice exhibited similar changes in their brown adipocyte lipid droplets under TN conditions. Correspondingly, the triglyceride content in BAT of *Txnip*^−/−^ mice significantly exceeded that in WT BAT (Fig. S1*H*).

To explore the effect of Txnip deficiency on BAT function, we determined the effects of acute cold stress on Txnip expression in the BAT of WT mice housed at room temperature (RT, 23 °C). Acute exposure to cold conditions (5 °C, for 6 h) resulted in a 2-fold increase in Txnip protein abundance relative to the baseline (Fig. 1*G*). Subsequent investigation into the impact of reduced Txnip levels on cold tolerance showed that both *Txnip*^−/−^ and *Txnip*^+/−^ mice, acclimated to TN conditions before acute cold exposure, exhibited a rapid decline in rectal temperatures, reaching life-threatening hypothermia at 3 h and 5 h, respectively (Fig. 1*H*). *Txnip*^−/−^ mice died within 4 to 6 h, while *Txnip*^+/−^ mice perished within 8 to 10 h of cold exposure, indicating a correlation between reduced *Txnip* copy number and deteriorated cold tolerance (Fig. 1*I*). Despite improved survival rates in WT mice housed at RT before cold exposure, *Txnip*^−/−^ mice could not be rescued, perishing within 12 h of cold exposure (Fig. S2, *A* and *B*). Furthermore, following a 5 h cold exposure, BAT from *Txnip*^−/−^ mice retained its enlarged and whitened appearance with most adipocytes exhibiting hypertrophic unilocular lipid droplets, whereas *Txnip*^+/−^ and WT BAT became smaller and brown appearance with varying degrees of multilocular lipid droplets (Fig. 1*J*). This observation was pronounced in mice acclimated to TN conditions but remained consistent in those maintained at RT (Fig. S2*C*). Functionally, thermographic imaging revealed a progressive decline in BAT surface temperatures in *Txnip*^−/−^ mice during acute cold exposure, suggesting that BAT of *Txnip*^−/−^ mice lacks the capability to generate heat in response to acute cold stress (Figs. 1, *K* and *L*, and S2, *D* and *E*).

### Txnip is essential for sustaining adaptive thermogenesis during acute cold stress

**Figure 2:**
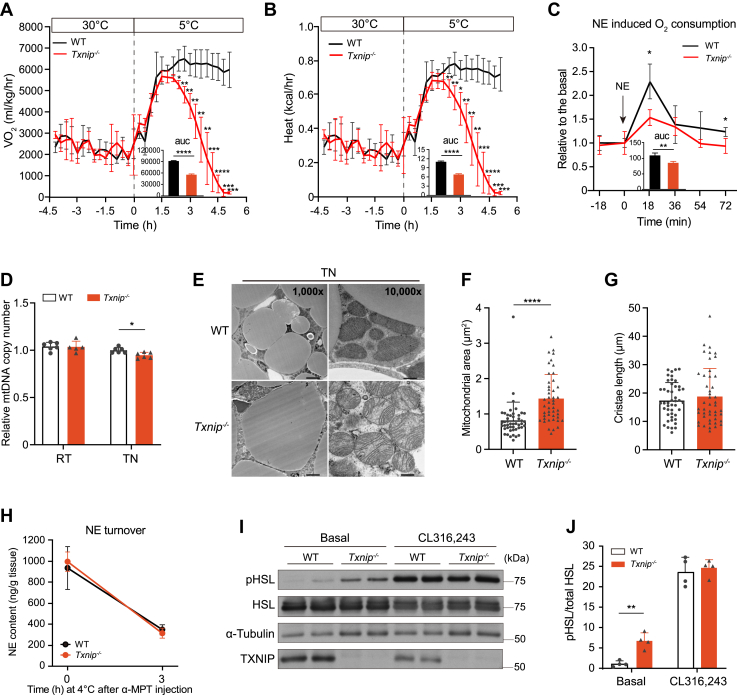
***Txnip* deficiency impairs metabolic respiration in response to acute thermogenic demand.***A*, O_2_ consumption rate (VO_2_) changes in WT and *Txnip*^*−/−*^ mice transitioning from TN to cold conditions (n = 4 per group). *B*, heat production in WT and *Txnip*^*−/−*^ mice transitioning from TN to cold conditions (n = 4 per group). *C*, relative VO_2_ in TN-acclimated WT and *Txnip*^*−/−*^ mice post intraperitoneal norepinephrine (NE) injection at 1 mg/kg (body weight) (n = 4 per group). *D*, relative mitochondrial DNA copy number in the BAT of WT and *Txnip*^*−/−*^ mice housed at RT and TN conditions (n = 6 per group). *E*, representative transmission electron microscopy (TEM) images of interscapular BAT from WT and *Txnip*^*−/−*^ mice acclimated to TN conditions for 1 week. The scale bar represents 0.5 μm. *F* and *G*, quantification of mitochondrial area (*F*) and total mitochondria cristae length (*G*) in BAT from WT and *Txnip*^*−/−*^ mice acclimated to TN conditions for 1 week (n = 46 mitochondria per group). *H*, NE turnover in BAT of WT and *Txnip*^*−/−*^ mice exposed to acute cold for 3 h after catecholamine synthesis inhibition with α-methyl-p-tyrosine (α-MPT) (200 mg/kg body weight, intraperitoneal injection). The mice were prehoused at RT (n = 6 per group). *I*, Western blots of hormone-sensitive lipase (HSL), phosphorylated HSL (pHSL), α-tubulin, and TXNIP in BAT lysates 5 min after injecting β3 agonist CL316,243 into WT and *Txnip*^*−/−*^ mice housed at TN conditions (n = 4 per group). *J*, pHSL quantitation from Western blot in (*I*). Data: mean ± SD. Statistical analysis: unpaired Student's *t* test (*A*–*D*, *F*, *G*, and *J*), two-way ANOVA with Bonferroni's *post hoc* test (*H*). ∗*p* < 0.05, ∗∗*p* < 0.01, ∗∗∗*p* < 0.001, and ∗∗∗∗*p* < 0.0001. See also Fig. S3. BAT, brown adipose tissue; pHSL, phosphorylated hormone-sensitive lipase; RT, room temperature; TN, thermoneutral; Txnip, thioredoxin-interacting protein.

Next, we examined the impact of *Txnip* deficiency on thermogenic function by recording whole-body parameters, such as O_2_ consumption rate (VO_2_), heat generation (Heat), and respiratory exchange ratio (RER). Surprisingly, under both TN and RT conditions, *Txnip*^*−/−*^ mice exhibited higher VO_2_ and Heat than WT mice, while their RER was lower during the light phase (Fig. S3, *A*–*I*). Upon acute transfer to cold conditions following TN acclimation, *Txnip*^−/−^ mice initially exhibited increased VO_2_ and Heat similar to WT mice. However, unlike WT mice, they failed to sustain this respiratory response, experiencing a rapid and profound decline in VO_2_ and Heat 1.5 h after transfer to cold conditions (Fig. 2, *A* and *B*). The norepinephrine (NE) challenge in TN-acclimated mice revealed a blunted increase in VO_2_ in *Txnip*^*−/−*^ mice compared to controls (Fig. 2*C*). To gain insights into defective metabolic respiration in *Txnip*^*−/−*^ mice, we examined mitochondrial abundance and morphology in BAT. A slight but significant decrease in mitochondrial DNA (mtDNA) copy number was detected in BAT of *Txnip*^*−/−*^ mice under TN conditions, but not at RT (Fig. 2*D*). The ultrastructure of BAT revealed hypertrophic lipid droplets and enlarged mitochondrial size in *Txnip*^−/−^ mice under TN conditions, while mitochondrial cristae length was indistinguishable between genotypes (Fig. 2, *E*–*G*). Subsequently, we assessed sympathetic activation and adrenergic receptor (AR) function in BAT. The NE turnover during cold exposure remained unchanged in BAT of *Txnip*^*−/−*^ mice (Fig. 2*H*). Regarding AR function, BAT of *Txnip*^*−/−*^ mice exhibited a significant baseline elevation of PKA activity assessed by detecting phosphorylated PKA substrates and hormone-sensitive lipase (HSL) (Figs.2, *I* and *J* and S3*J*). Furthermore, the induction of phosphorylated PKA substrates and HSL in *Txnip*^*−/−*^ BAT by CL316,243, a β3-AR agonist, mirrored that in WT BAT. These observations implied that sympathetic activation and AR function are preserved in BAT of *Txnip*^*−/−*^ mice.

**Figure 3:**
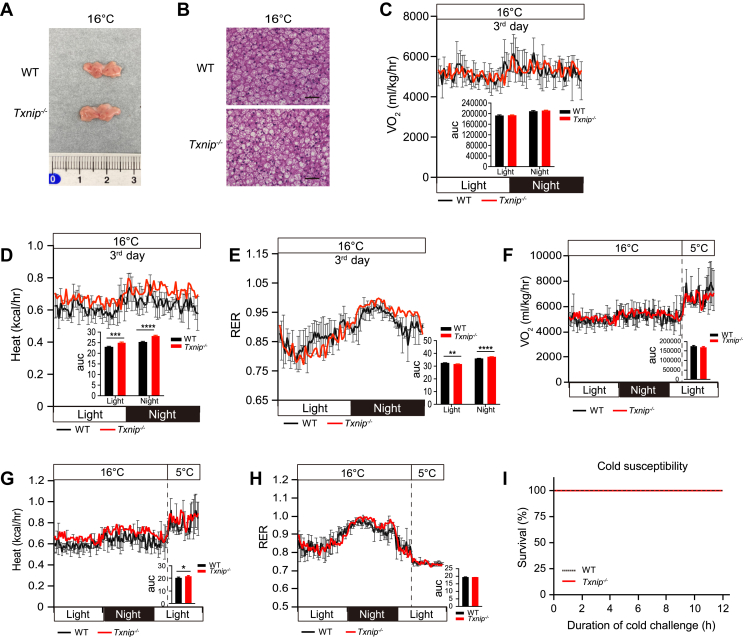
**Chronic cold exposure protects *Txnip***^***−/−***^**mice from lethal hypothermia in cold circumstances.***A*, representative interscapular BAT image from WT and *Txnip*^−/−^ mice acclimated to 16 °C for 1 week. *B*, H&E staining of interscapular BAT from WT and *Txnip*^−/−^ mice acclimated to 16 °C for 1 week. The scale bar represents 50 μm; *C*–*E*, VO_2_ (*C*), heat production (*D*), and RER (*E*) in WT and *Txnip*^−/−^ mice on the third day of 16 °C housing (n = 3, 4). *F*–*H*, adaptive changes of VO2 (*F*), heat production (*G*), and RER (*H*) in WT and *Txnip*^−/−^ mice transitioning from 16 °C to cold exposure at 5 °C. Mice were exposed to cold for 8 h after prehousing at 16 °C for 1 week (n = 3, 4). *I*, survival rate of WT and *Txnip*^−/−^ mice exposed to cold after prehousing at 16 °C for 1 week (n = 9 per group). Data: mean ± SD. Statistical analysis: unpaired Student's *t* test (*C–H*), log-rank (Mantel–Cox) test (*I*). ∗*p* < 0.05, ∗∗*p* < 0.01, ∗∗∗*p* < 0.001, and ∗∗∗∗*p* < 0.0001. BAT, brown adipose tissue RER, respiratory exchange ratio; Txnip, thioredoxin-interacting protein;.

We further examined the thermogenic capability of *Txnip*^*−/−*^ mice after acclimating them to 16 °C for 7 days, exposing them to chronic mild cold stress. Surprisingly, BAT of *Txnip*^*−/−*^ mice exhibited similar morphology to WT BAT, with brown adipocytes displaying multilocular small lipid droplets (Fig. 3, *A* and *B*). With this morphological reversal, *Txnip*^*−/−*^ mice exhibited similar VO_2_ and higher heat generation during mild cold stress (16 °C), with a lower RER during the light phase and a higher RER at night, indicating enhanced fuel utilization in *Txnip*^*−/−*^ mice (Fig. 3, *C*–*E*). Furthermore, *Txnip*^*−/−*^ mice acclimated to 16 °C became resistant to lethal cold stress, showing restored thermogenic response (Fig. 3, *F*–*I*). These results highlight the essential role of Txnip in thermogenesis during acute cold stress.

### Txnip maintains basal expression of BAT signature genes

To explore the underlying cause of thermogenic defects, we examined BAT in younger mice grown at RT. In 5-week-old *Txnip*^*−/−*^ mice, BAT mass surpassed that of WT mice, with increased unilocular lipid droplets (Fig. S4, *A* and *B*). These mice also showed enlarged inguinal white adipose tissue (iWAT) and hypertrophic adipocytes (Fig. S4, *C* and *D*). The adipocyte marker *Adipoq* expressed similarly between WT and *Txnip*^−/−^ mice adipose tissues, while the lipogenesis-related gene *Scd1* was significantly upregulated in both BAT and iWAT of *Txnip*^−/−^ mice, indicating increased lipogenic activity in *Txnip*-deficient adipose tissues (Fig. S4*E*). While *Ucp1* and *Dio2* were downregulated, other browning signature genes—including *Pgc-1α*, *Prdm16*, *Cidea*, and *Pparα*—appeared maintained in *Txnip*^*−/−*^ BAT, suggesting normal maturation of brown adipocytes in the absence of *Txnip* (Fig. S4*E*).

**Figure 4:**
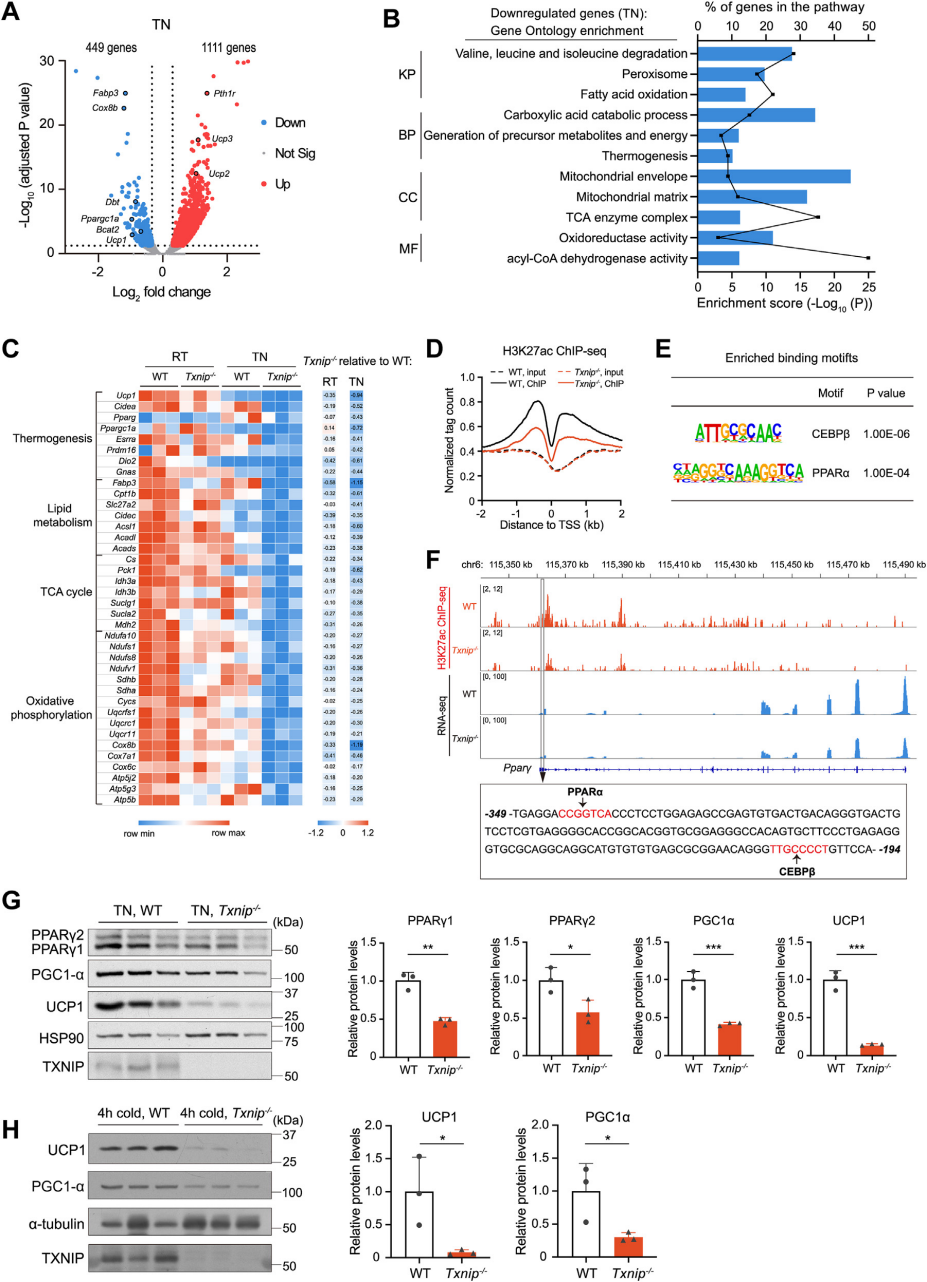
***Txnip* maintains basal thermogenic and metabolic gene expression in BAT.***A*, volcano plot of RNA-seq data showing *Txnip*-regulated BAT genes in *Txnip*^*−/−*^*versus* WT mice acclimated to TN (fold change >1.25 up (*red*) or <0.8 down (*blue*)) (n = 3 per group). *B*, gene ontology and pathway analysis of downregulated genes in *Txnip*^*−/−*^ BAT under TN conditions, identified by RNA-seq and selected based on the enrichment score. The bars represent the enrichment scores for the downregulated pathways. The *lines* represent the number of downregulated genes as a percentage of the corresponding pathway. *C*, *left:* heat map of selected BAT signature genes in WT and *Txnip*^*−/−*^ mice housed at RT or TN conditions. *Right:* heat map of selected BAT signature genes in *Txnip*^*−/−*^*versus* WT mice housed at RT or TN conditions. *D*, H3K27ac ChIP-seq profiles within ±2 kb of the transcription start sites of genes in BAT isolated from WT and *Txnip*^*−/−*^ mice at TN conditions. *E*, enriched known motifs at sites with decreased H3K27ac binding in BAT from *Txnip*^*−/−*^*versus* WT mice at TN conditions. *F*, *upper:* genome browser tracks displaying *Pparγ* loci with ChIP-seq and RNA-seq data under TN conditions. *Bottom:* DNA sequence of the 155 bp in the proximal promoter that displayed reduced H3K27ac ChIP signals in *Txnip*^*−/−*^ BAT. The binding sites of PPARα and CEBPβ are highlighted in *red letters*. *G*, Western blot and quantitation of PPARγ, PGC-1α, and UCP1 in BAT from WT and *Txnip*^*−/−*^ mice housed at TN conditions. HSP90 was used as the loading control (n = 3 per group). *H*, Western blot and quantitation of UCP1 and PGC-1α in BAT from WT and *Txnip*^*−/−*^ mice housed at TN conditions for 1 week, followed by 4-h acute cold exposure at 5 °C. α-Tubulin was used as the loading control (n = 3 per group). Data: mean ± SD. Statistical analysis: Unpaired Student's *t* test (*G* and *H*). ∗*p* < 0.05, ∗∗*p* < 0.01, ∗∗∗*p* < 0.001, and ∗∗∗∗*p* < 0.0001. See also Figs. S4–S6. BAT, brown adipose tissue; BP, biological process; CC, cellular component; ChIP-seq, chromatin immunoprecipitation sequencing; KP, KEGG pathway; MF, molecular function; TN, thermoneutral; Txnip, thioredoxin-interacting protein; Ucp1, uncoupling protein 1.

To investigate the molecular mechanisms by which Txnip controls the thermogenic capacity of BAT, we compared BAT signature genes in *Txnip*^*−/−*^ mice and their WT littermates housed at RT or TN conditions using RNA-seq. Under RT conditions, 562 genes were dysregulated in *Txnip*^*−/−*^ mice (291 upregulated and 271 downregulated) (Fig. S5*A*). A more distinct pattern emerged under TN conditions, with 1560 expression changes observed, among which 1111 genes were upregulated and 449 downregulated (Fig. 4*A*). For metabolic genes, *Fabp3* and *Cox8b* displayed significant downregulation among the genes affected at TN conditions, followed by *Dbt*, *Ppargc1a* (ecoding PGC-1α), *Bcat2*, and *Ucp1* (Fig. 4*A*). Interestingly, while *Ucp1*, crucial for canonical thermogenesis, showed downregulation, other genes related to alternative pathways like *Pthr1*, *Ucp2*, and *Ucp3*, exhibited upregulation in *Txnip*^*−/−*^ BAT. Gene Ontology analysis of all significantly downregulated genes in *Txnip*^*−/−*^ BAT revealed prevalent dysregulation in pathways such as branched-chain amino acid (BCAA) degradation, peroxisome, fatty acid (FA) oxidation, carboxylic acid catabolic process, mitochondrial membrane, and mitochondrial matrix (Figs. 4*B* and S5*B*). Most key genes involved in BAT thermogenesis, lipid metabolism, the tricarboxylic acid (TCA) cycle, and oxidative phosphorylation were dysregulated in BAT of *Txnip*^*−/−*^ mice under both RT and TN conditions (Fig. 4*C*). Notably, these genes showed more pronounced downregulation under TN conditions than at RT (Fig. 4*C*), as confirmed by reverse transcription qPCR analysis (Fig. S5, *C*–*H*). Interestingly, under mild cold-acclimated conditions (16 °C), the expression of most thermogenic genes in BAT of *Txnip*^*−/−*^ mice remained unaffected, except for *Ucp1* and *Dio2* (Fig. S5*I*).

Despite limited knowledge regarding the role of Txnip in transcriptional regulation, we investigated the correlation between transcription profiles and the epigenome within BAT of *Txnip*^*−/−*^ mice under basal conditions, using chromatin immunoprecipitation followed by sequencing (ChIP-seq) targeting acetylated histone H3 lysine 27 (H3K27ac) as a marker for active enhancers (27). The analysis revealed 7468 increased and 6028 decreased ChIP-seq signals in BAT of *Txnip*^*−/−*^ mice relative to WT (Fig. S6, *A* and *B*). Most of the altered ChIP signals were distributed across intergenic and intronic regions. However, the reduced H3K27ac levels in BAT of *Txnip*^*−/−*^ mice were specifically localized within the proximal regions of the transcription start site (Figs. 4*D* and S6*C*). Furthermore, the *de novo* motif analysis revealed an enrichment of binding motifs associated with known transcription factors, specifically CEBPβ and PPARα within the regions exhibiting decreased H3K27ac levels (Fig. 4*E*). To obtain epigenetic insights into altered thermogenic properties within BAT of *Txnip*^*−/−*^ mice, we analyzed H3K27ac occupancy on the *Pparγ* loci, a critical regulator of BAT thermogenesis (28). Txnip deficiency reduced H3K27ac ChIP signals in the proximal promoter that contained CEBPβ- and PPARα-binding sites as well as in a distal enhancer, leading to the attenuation of *Pparγ* transcription (Fig. 4*F*). Consistently, *Pgc-1α*, a *Pparγ* coactivator, also showed reduced ChIP signals and transcription in *Txnip*^*−/−*^ BAT under TN conditions (Fig. S6*D*). However, H3K27ac signals in the *Ucp1* loci, the transcription target of Pparγ and Pgc-1α, did not decrease but instead increased in an enhancer region of −2.4 kbp from the transcription start site in BAT of *Txnip*^*−/−*^ mice (Fig. S6*E*). Nevertheless, *Ucp1* transcription notably declined, indicating the importance of the paucity of *trans-*acting factors, such as Pparγ/α, Pgc-1α, and PR/SET domain 16 (Prdm16) (29, 30, 31), in suppressing *Ucp1* due to Txnip loss. Accordingly, we detected a significant decrease in the protein expression of PPARγ, PGC-1α, and UCP1 in BAT of *Txnip*^*−/−*^ mice under TN conditions (Fig. 4*G*). Additionally, expression of UCP1 and PGC-1α remained suppressed in BAT of *Txnip*^−/−^ mice during the acute cold exposure, whereas WAT rather upregulated *Ucp1* with maintaining *Pgc-1α* (Figs. 4*H* and S6, *F*–*I*). These findings support a pivotal role for Txnip in driving the basal expression of thermogenic signature genes in BAT.

### *Txnip*-deficient mice fail to activate BAT oxidative metabolism in response to acute cold stress

**Figure 5:**
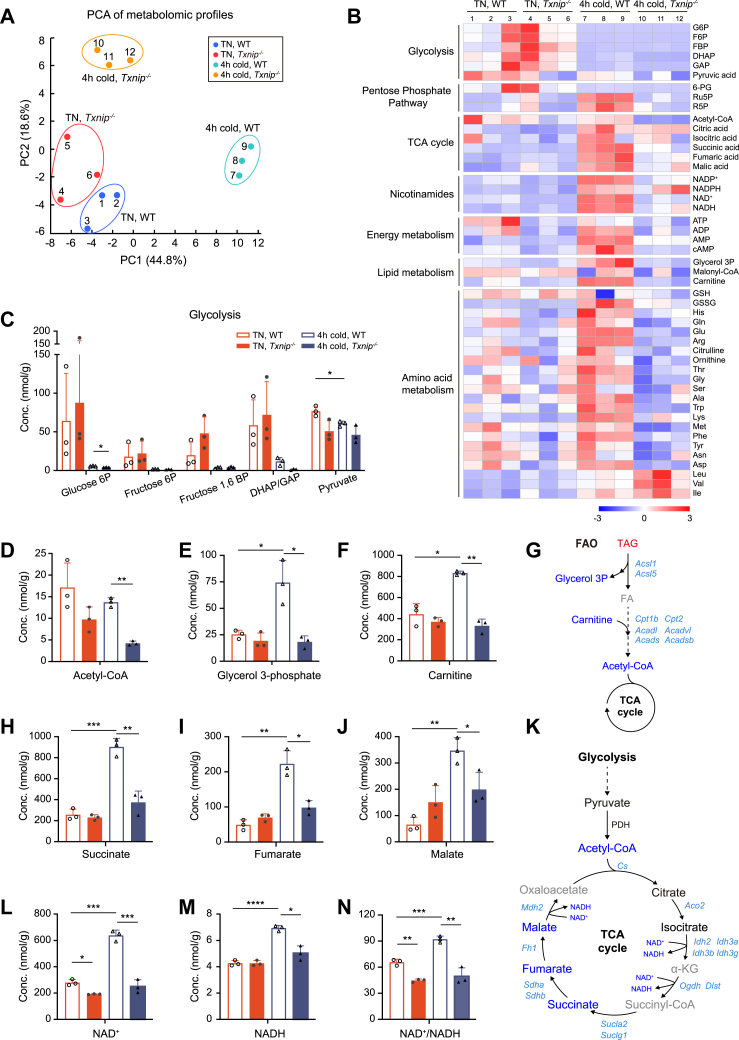
**Metabolomic profile alterations in BAT of *Txnip***^***−/−***^**mice in response to acute cold stress.***A*, principal component analysis (PCA) of the metabolomic data in BAT from WT and *Txnip*^*−/−*^ mice housed in TN conditions for 1 week, followed by 4-h cold exposure at 5 °C (n = 3 per group). *B*, heat map analysis of the selected metabolites in BAT from WT and *Txnip*^*−/−*^ mice housed at TN conditions for 1 week, followed by 4-h cold exposure at 5 °C (n = 3 per group). *C*–*F*, levels of glycolysis intermediates (*C*), acetyl-CoA (*D*), glycerol 3-phosphate (*E*), and carnitine (*F*) in BAT of WT and *Txnip*^*−/−*^ mice housed at TN conditions for 1 week, followed by 4-h cold exposure at 5 °C (n = 3 per group). *G*, changes in fatty acid oxidation (FAO)-related metabolites and genes. *Light blue:* downregulated genes in *Txnip*^*−/−*^*versus* WT BAT at TN conditions; *red:* increased metabolite levels in *Txnip*^*−/−*^*versus* WT BAT after 4-h of cold exposure; *blue:* decreased metabolite levels in *Txnip*^*−/−*^*versus* WT BAT after 4-h of cold exposure; *gray:* undetectable metabolites. *H*–*J*, levels of TCA cycle-related organic acids in BAT of WT and *Txnip*^*−/−*^ mice housed at TN conditions for 1 week, followed by 4-h cold exposure at 5 °C (n = 3 per group). *K*, changes in TCA cycle-related metabolites and genes. *Light blue:* downregulated genes in *Txnip*^*−/−*^*versus* WT BAT at TN conditions; *blue:* decreased metabolite levels in *Txnip*^*−/−*^*versus* WT BAT after 4-h of cold exposure; *black:* unchanged metabolites between WT and *Txnip*^*−/−*^ BAT after 4-h of cold exposure; *gray:* undetectable metabolites. *L–N*, levels of NAD^+^ (*L*), NADH (*M*), and NAD^+^/NADH (*N*) in BAT of WT and *Txnip*^*−/−*^ mice housed at TN conditions for 1 week, followed by 4-h cold exposure at 5 °C (n = 3 per group). Data: mean ± SD. Statistical analysis: unpaired Student's *t* test (*C–F*, *H–J*, and *L–N*). ∗*p* < 0.05, ∗∗*p* < 0.01, ∗∗∗*p* < 0.001, and ∗∗∗∗*p* < 0.0001. See also Fig. S7. BAT, brown adipose tissue; TCA, tricarboxylic acid; TN, thermoneutral; Txnip, thioredoxin-interacting protein.

To investigate the metabolic mechanism underlying blunted thermogenesis due to Txnip loss, we analyzed the BAT metabolome in *Txnip*^*−/−*^ and WT mice acclimated to TN conditions as well as in those exposed to acute cold (5 °C, 4 h). The principal component analysis showed clear intragroup clustering and notable divergence between *Txnip*^*−/−*^ and WT BAT, particularly under acute cold stress (Fig. 5*A*). Figure 5*B* illustrates that in response to acute cold stress, WT BAT exhibited increased levels of most metabolites related to metabolic pathways and processes other than glycolysis. However, this response was blunted in BAT of *Txnip*^*−/−*^ mice. In contrast, glycolysis-related metabolites decreased in both WT and *Txnip*^*−/−*^ BAT during cold stress (Fig. 5*C*). The concentration of pyruvate and the phosphorylation of pyruvate dehydrogenase E1a remained unchanged despite *Txnip* deficiency (Fig. S7*A*), indicating that glycolytic flux into the TCA cycle persisted in BAT of *Txnip*^*−/−*^ mice. Meanwhile, blood glucose levels declined to approximately 100 mg/dl in *Txnip*^*−/−*^ mice during cold exposure, while remaining steady in WT mice, indicating a potential increase in circulating glucose uptake by *Txnip*^*−/−*^ mice (Fig. S7*B*). Despite maintaining glycolysis, BAT of *Txnip*^*−/−*^ mice exhibited a significant decrease in acetyl-CoA under cold exposure compared to WT, suggesting attenuated mobilization of other nutritional substrates (Fig. 5*D*). While WT BAT increased glycerol 3-phosphate and carnitine levels in response to cold stress, this response was completely abolished in BAT of *Txnip*^*−/−*^ mice (Fig. 5, *E* and *F*). Considering this observation along with the decreased baseline transcription of lipid metabolism genes (Fig. 4*C*), BAT of *Txnip*^*−/−*^ mice likely failed to activate FA oxidation in response to acute cold stress (Fig. 5*G*). A decline in acetyl-CoA was followed by a significant reduction in the intermediate substrates of the TCA cycle—succinate, fumarate, and malate—along with attenuated baseline transcription of several enzymes involved in this cycle (Fig. 5, *H*–*K*). Importantly, the elevation of NAD+, NADH, and the NAD+/NADH ratio in response to acute cold stress was abolished in BAT of *Txnip*^*−/−*^ mice (Fig. 5, *L*–*N*), indicating that BAT of *Txnip*^*−/−*^ mice failed to activate oxidative metabolism crucial for thermogenesis during acute cold stress.

### *Txnip* deficiency impairs BCAA catabolism in BAT

**Figure 6:**
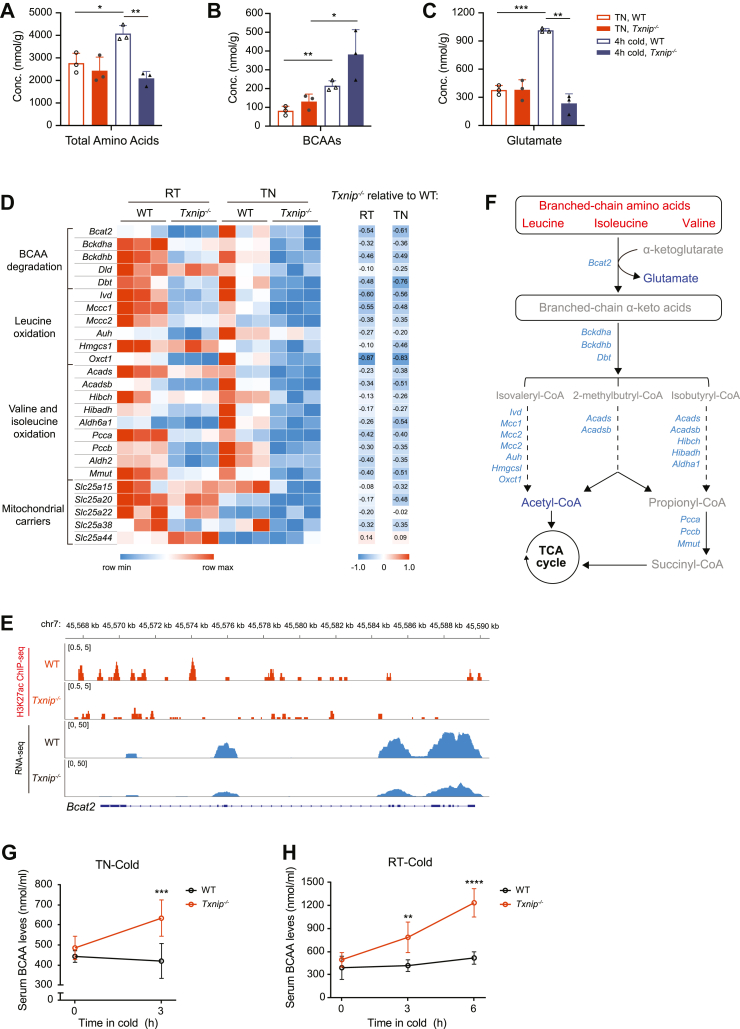
***Txnip* deficiency impairs BAT BCAA catabolism in response to acute cold stress.***A*–*C*, amino acid levels (*A*), total BCAAs (*B*), and glutamate (*C*) in BAT of TN-acclimated WT and *Txnip*^*−/−*^ mice for 1 week, followed by 4-h cold exposure at 5 °C (n = 3 per group). *D*, *left:* heat map of BCAA oxidation genes in BAT from WT and *Txnip*^*−/−*^ mice housed at RT or TN conditions. *Right:* heat map of BCAA oxidation genes in BAT from *Txnip*^*−/−*^ mice relative to WT mice housed at RT or TN. *E*, genome browser tracks displaying *Bcat2* loci with ChIP-seq and RNA-seq data under TN conditions. *F*, changes in BCAA catabolism–related metabolites and genes. *Light blue:* downregulated genes in *Txnip*^*−/−*^*versus* WT BAT at TN conditions; *red:* increased metabolite levels in *Txnip*^*−/−*^*versus* WT BAT after 4-h of cold exposure; *blue:* decreased metabolite levels in *Txnip*^*−/−*^*versus* WT BAT after 4-h of cold exposure; *gray:* undetectable metabolites. *G*, changes in serum BCAA levels in WT and *Txnip*^*−/−*^ mice during acute cold exposure after TN acclimation (n = 5 per group). *H*, changes in serum BCAA levels in WT and *Txnip*^*−/−*^ mice during acute cold exposure after RT acclimation (n = 7, 8). Data: mean ± SD. Statistical analysis: unpaired Student's *t* test (*A–C*), two-way ANOVA with Bonferroni's *post hoc* test (*G* and *H*). ∗*p* < 0.05, ∗∗*p* < 0.01, ∗∗∗*p* < 0.001, and ∗∗∗∗*p* < 0.0001. See also Fig. S8. BAT, brown adipose tissue; BCAA, branched-chain amino acid; ChIP-seq, chromatin immunoprecipitation sequencing; RT, room temperature; TN, thermoneutral; Txnip, thioredoxin-interacting protein.

Amino acids serve as substrates in various metabolic pathways. While most measured amino acids increased in the BAT of WT mice under acute cold stress, this response was blunted in *Txnip*^*−/−*^ mice (Fig. 5*B*). This was further confirmed by assessing total amino acid levels in BAT (Fig. 6*A*). Interestingly, although no significant change was observed between genotypes under TN acclimation, valine, leucine, and isoleucine levels appeared to increase in BAT of *Txnip*^*−/−*^ mice after acute cold exposure (Fig. 5*B*). Their total amounts, measured as BCAAs, increased approximately 2-fold in *Txnip*^*−/−*^ mice relative to WT mice after acute cold exposure (Fig. 6*B*). BCAAs are energetically more efficient than other amino acids and are actively catabolized in BAT for thermogenesis during cold exposure (32). We observed that while glutamate levels, generated at the initial step of the BCAA catabolism, markedly increased in WT BAT in response to acute cold stress without a concurrent increase in GSH levels, this response was completely abolished in BAT of *Txnip*^*−/−*^ mice (Figs. 6*C* and S8*A*). Furthermore, the baseline transcription of enzymes and transporters involved in BCAA catabolism was downregulated in BAT of *Txnip*^*−/−*^ mice, correlating with epigenetic modifications in the *Bcat2* loci (Figs. 6, *D* and *E* and S8*B*). Thus, these findings indicate the impaired BAT activity of *Txnip*^*−/−*^ mice to facilitate BCAA catabolism, especially in response to acute thermogenic requirements (Fig. 6*F*). Moreover, while cold-activated BAT promotes systemic BCAA clearance in both humans and mice (32), *Txnip*^*−/−*^ mice exhibited a progressive increase in circulating BCAA levels during acute cold exposure (Fig. 6, *G* and *H*). This implies a potential attenuation in the clearance of circulating BCAAs associated with their utilization in BAT.

### A ketogenic diet exacerbates cold intolerance in *Txnip*^*−/−*^ mice without developing hypoglycemia

**Figure 7:**
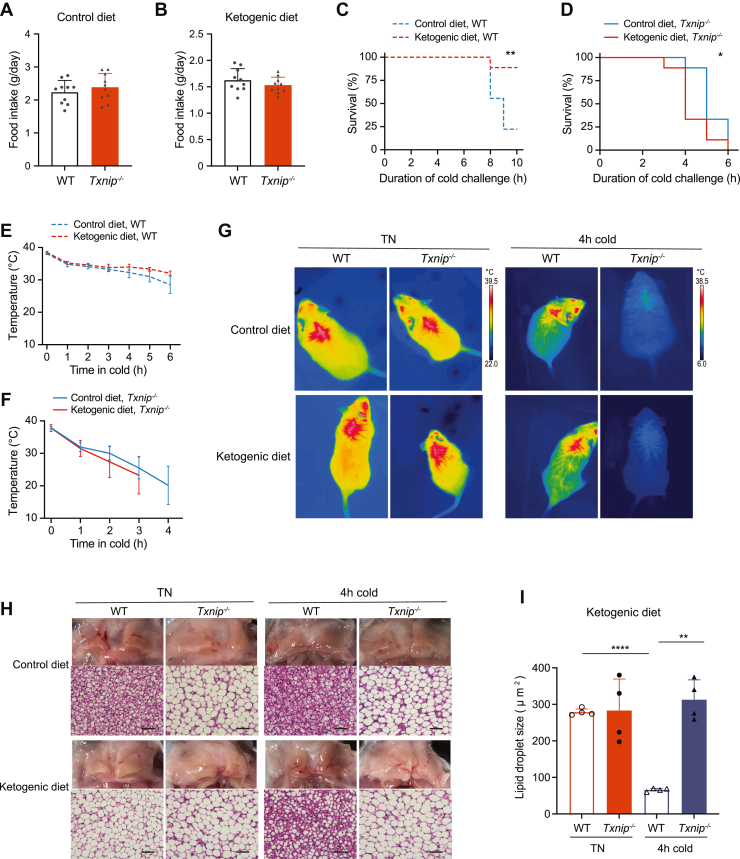
**Ketogenic diet fails to restore cold intolerance in *Txnip***^***−/−***^**mice.***A* and *B*, food intake for control diet (*A*) and ketogenic diet (*B*) in WT and *Txnip*^*−/−*^ mice housed at TN conditions (n = 10 per group). *C* and *D*, survival rates of WT (*C*) and *Txnip*^*−/−*^ (*D*) mice during acute cold exposure after feeding ketogenic and control diets for 1 week under TN conditions (n = 9 per group). *E* and *F*, interscapular BAT surface temperatures of WT (*E*) and *Txnip*^*−/−*^ (*F*) mice during acute cold exposure after feeding ketogenic and control diets for 1 week under TN conditions (n = 4 per group). *G* and *H*, representative infrared images (*G*), macroscopic and H&E staining images (*H*) of WT and *Txnip*^*−/−*^ mice fed a control diet and ketogenic diet for 1 week under TN conditions, followed by 4-h of acute cold exposure. *I*, quantification of lipid droplet size from BAT H&E-stained slides in (*H*) (n = 4 per group). Data: mean ± SD. Statistical analysis: unpaired Student's *t* test (*A*, *B*, and *I*), log-rank (Mantel–Cox) test (*C* and *D*), two-way ANOVA with Bonferroni's *post hoc* test (*E* and *F*). ∗*p* < 0.05, ∗∗*p* < 0.01, ∗∗∗*p* < 0.001, and ∗∗∗∗*p* < 0.0001. BAT, brown adipose tissue; TN, thermoneutral; Txnip, thioredoxin-interacting protein.

A ketogenic diet (KG) stimulates metabolic conditions akin to fasting, inducing a metabolic shift in fuel preference from glucose to FAs or ketone bodies. A recent study has shown that a KG corrected mitochondrial function in the BAT of a specific mouse line with *Txnip* deficiency (33). Therefore, we tested the effects of a KG on BAT thermogenesis and cold intolerance in *Txnip*^*−/−*^ mice. Because *Txnip*^−/−^ mice reportedly have susceptibilities to fasting, we used a modified KG in which the carbohydrate proportion was increased to protect the mice (Table S1). Although food intake for the control diet and ketogenic did not differ between WT and *Txnip*^*−/−*^ mice housed at TN conditions, upon acute cold exposure, WT fed a modified KG significantly improved the survival rate during the observation period (Fig. 7, *A*–*C*). In contrast, despite no hypoglycemic development, *Txnip*^*−/−*^ mice fed a modified KG died significantly earlier than those fed control diet (Fig. 7*D*). Consistently with the survival rates, a modified KG slowed a decline in BAT surface temperature during the cold exposure in WT but had the opposite effect in *Txnip*^−/−^ mice (Fig. 7, *E*–*G*). Notably, WT and *Txnip*^−/−^ mice similarly displayed BAT whitening with enlarged unilocular lipid droplets upon TN acclimation with KG feeding (Fig. 7, *H* and *I*). Nevertheless, under the cold stress, the lipid droplets in WT BAT became smaller and morphologically reverted to multilocular, whereas those in BAT of *Txnip*^−/−^ mice showed no distinct changes, indicating its inability to activate utilization of FAs for thermogenesis.

## Discussion

The tight regulation of metabolic and thermogenic activities is pivotal for maintaining core body temperature in mammals. Although several studies have delved into the transcriptional networks governing thermogenic genes within brown adipocytes, many aspects of this process remain incompletely understood. This study identified Txnip as a crucial factor in preserving brown adipocyte identity and ensuring preparedness for acute and life-threatening cold stress. *Txnip*-deficient mice exhibited BAT whitening along with decreased transcription of BAT signature and metabolic genes. Upon exposure to acute cold stress, the BAT of *Txnip*^*−/−*^ mice failed to activate oxidative metabolism in BCAA and FAs, rendering them highly susceptible to severe cold stress. Our findings underscore that Txnip plays a role in maintaining BAT identity and is essential for ensuring BAT thermogenic function in response to acute cold stress, providing further insights into inducible thermogenesis under acute cold stress.

The previous studies have indicated that *Txnip*^*−/−*^ mice are intolerant to starvation, inflammation, and endurance exercise (5, 7, 15), suggesting an integral role of Txnip in protection against various physiological stresses. In this context, our study uncovers the impact of reduced Txnip expression on thermogenesis, specifically in response to acute cold stress. This finding not only enhances our understanding of mammalian biology but also indicates that Txnip plays a crucial role in enabling mice to adapt to environmental changes, potentially supporting their nocturnal activity during evolution.

Txnip expression was rapidly declined in BAT by administration with CL316,243, whereas it conversely increased following a 6-h cold exposure. This difference might be attributed to distinct adrenergic stimulation and/or different time course. Adrenergic stimulation mobilizes nutritional substrates for thermogenic metabolism. In this context, reducing Txnip—which is a negative regulator of glucose uptake (5, 16)—is reasonable for brown adipocytes to facilitate glucose utilization in the early response to adrenergic stimulation. Meanwhile, it is likely that the cells upregulate Txnip to maintain redox homeostasis when ROS is excessively generated along activation of oxidative metabolism. However, future studies examining how Txnip expression is regulated under acute cold stress are needed.

It has been shown that loss of Txnip enhanced insulin secretion and insulin-mediated glucose uptake, facilitating glucose disposal (5, 16). Given the general understanding, whereas the effects of *Txnip* deficiency on glucose metabolism and BAT function are seemingly contradictory, the recent study that examined the different line of *Txnip*-deficient mice has suggested a possibility that increased glucose influx affects mitochondrial integrity and electron transport chain efficiency in brown adipocytes (33). The metabolomic profile of *Txnip*^−/−^ BAT indicated enhanced glycolysis, similar to WT BAT, in response to acute cold stress. This supposedly accounts for preservation of the early elevation of O2 consumption rate (VO2) in *Txnip*^−/−^ mice. However, the mice could not maintain potentiation of metabolic respiration, and their VO2 rapidly declined. This failure might be attributed to the severely blunted catabolic responses of FAs and BCAAs in BAT of *Txnip*^−/−^ mice, hindering its ability to maintain thermogenic metabolism. Starvation, endurance exercise, and cold stress, all are the conditions under which mice require Txnip for a metabolic shift from glucose to other nutrients. Hence, our observations provide further insights into coordinating fuel selection for adaption to various physiological stresses.

*Txnip*^−/−^ mice were severely susceptible to acute cold exposure, whereas they were capable of increasing thermogenesis as housing temperatures decreased. This suggests that physiological thermogenic mediators are not critically impaired in *Txnip*^−/−^ mice. In addition, RER recorded in *Txnip*^−/−^ mice significantly decreased during the light phase relative to that recorded in WT, indicating an increase in systemic FA oxidation. Given that thermogenic metabolism in BAT would be blunted by lacking Txnip, other thermogenic organs, such as WAT, where FAs are preferentially utilized, might retain or even potentiate the basal thermogenesis. With a relevance of this observation, WAT showed increased *Ucp1* expression during the acute cold exposure, while this response was totally eliminated in BAT of *Txnip*^−/−^ mice. Collectively, our observations indicate that Txnip plays a pivotal role in enabling BAT to adaptively activate its thermogenic program when suddenly exposed to life-threatening cold conditions. Although the precise function of Txnip in brown adipocytes remains unclear, a recent report has demonstrated that oxidative function in isolated mitochondria from BAT of a distinct line of *Txnip*-deficient mice was impaired (33). This experimental evidence may, at least partly, explain our observations on BAT. However, to further understand a cell-autonomous role of Txnip in BAT, future studies exploring the effects of brown adipocyte–specific deletion of Txnip will be needed.

*Txnip* deficiency leads to the downregulation of basal transcription in BAT signature and metabolic genes. We observed a correlation between the downregulation of key regulators, such as *Pparγ* and *Pgc-1α*, critical for BAT thermogenic transcription network and reduced H3K27ac levels on these genes. However, the underlying mechanisms regulated by Txnip remain unclear. A recent report described the nuclear distribution of Txnip in mouse brown adipocytes (34), suggesting a potential nuclear role for Txnip. In addition, the observed upregulation of Txnip in BAT following acute cold exposure raises questions about its increased nuclear expression or facilitated nuclear translocation under cold stress. Consequently, future studies should explore the nuclear function of Txnip in regulating BAT thermogenic gene transcription and its nuclear expression under cold stress.

Intriguingly, in the BAT of *Txnip*^*−/−*^ mice, the transcription of *Ucp1*, *Pparγ*, and *Pgc-1α* was notably lower at TN conditions than at RT, indicating that the loss of Txnip has a more pronounced effect on the basal expression of these genes than the absence of sympathetic stimulation. This lowered transcription was also observed in several other genes critical for oxidative phosphorylation and FA and BCAA metabolism. Thus, Txnip plays a critical role in maintaining the basal expression of *Ucp1* and other genes regulating oxidative and fuel metabolism in BAT. Our findings underscore the physiological role of Txnip in preserving thermogenesis in response to acute exposure to life-threatening cold temperatures. Txnip plays a unique role in priming brown adipocytes to promptly support thermogenic respiration and immediate heat production. Thus, unlike chronic cold stress-induced thermogenic mechanisms that require time to evolve, Txnip establishes a basal thermogenic tone, enabling BAT to rapidly respond to acute, life-threatening cold exposures.

In conclusion, our study reveals a novel physiological role of Txnip in BAT adaptive thermogenesis in response to acute cold stress. With the involvement of Txnip in various metabolic organs, it emerges as a critical regulator of energy metabolism, adapting to environmental stresses, such as starvation and cold exposure. Therefore, our findings contribute to understanding the physiological basis of responding to acute cold stress and shed light on mammalian biology.

## Experimental procedures

### Animals

Animal experiments adhered to the ethical regulations and protocols approved by the Institutional Animal Care and Use Committee of Yamaguchi University School of Medicine. Mice were housed in a specific-pathogen–free and climate-controlled facility at 23 °C under a 12-h light/dark cycle with standard chow (Oriental yeast) and water ad libitum. For KG studies, mice were fed either a modified KG or a control diet from 10 weeks of age for 1 week. The diet compositions used in this study are presented in Table S1. For thermoneutrality studies, mice were acclimated to 30 °C for 1 week in a climate-controlled animal chamber (Shin Factory, HC-10). *Txnip*^*−/−*^ mice were generated as previously described (35) and maintained on a C57B/6J background, while *Txnip*^*+/−*^ mice were obtained by crossing C57B/6J with *Txnip*^*−/−*^ mice. All experiments were performed on male age-matched mice (9–12 weeks old), unless stated otherwise. Mice of identical genotypes were randomly allocated to different treatment groups without blinding.

### Cold tolerance test

For acute cold exposure, mice were acclimated to 30 °C or 23 °C for 1 week before being exposed to cold (5 °C) and then individually housed in prechilled cages with bedding and free access to water but no food. The core body temperature of the mice was monitored hourly for prehousing at 30 °C or every 2 h for prehousing at 23 °C using a rectal probe (A&D Company). For chronic cold exposure, mice were acclimated to 16 °C for 1 week and then transferred to cold (5 °C). A core body temperature of ≤10 °C was considered an event for survival analysis.

### Infrared thermography

Infrared images of the mice were captured using a handheld thermal camera (FLIR E53). To expose the BAT surface area, a small amount of Vaseline was applied between the shoulder blades of the mice to brush up the neck fur before capturing the images. The BAT surface temperature was determined using FLIR Tools version 6 (freely available at https://flir.custhelp.com).

### Whole-animal O_2_ consumption rate and indirect calorimetry

Whole-animal O_2_ consumption rate (VO_2_) and CO2 production rate (VCO_2_) were measured, and indirect calorimetry was performed using an Oxymax apparatus (Columbus Instruments) equipped with a temperature-controlled animal chamber. Ten-week-old mice were initially monitored and maintained at 23 °C for 3 days, followed by 30 °C for 7 days before exposure to a cold challenge at 5 °C. For assessment under chronic cold conditions, mice were monitored and maintained at 23 °C for 3 days and then exposed to 16 °C for 7 days before the cold challenge at 5 °C. For the NE challenge, mice acclimated to 30 °C for 1 week were intraperitoneally injected with NE (Sigma-Aldrich) at 1 mg/kg body weight and immediately returned to their cages, continuously monitoring VO_2_ until the rates declined. The VO_2_ measurements were performed every 18 min for each mouse throughout the experiment, with the VO_2_ data normalized to body weight. The presented data include measurements from the final day of each condition and the first day of the condition transition. The theoretical value of heat production, denoted as Heat, was calculated using the formula (3.815 + 1.232 × RER) × VO_2_.

### Transmission electron microscopy

For transmission electron microscopy sample collection, cardiac perfusion was performed under anesthesia with a fixative buffer (2% glutaraldehyde, 2% paraformaldehyde in 0.1 M phosphate buffer). Interscapular BAT was dissected, cut into 1 mm pieces, and fixed in the fixative buffer (2% glutaraldehyde, 2% paraformaldehyde in 0.1 M phosphate buffer) overnight at 4 °C. Samples were rinsed in 0.1 M phosphate buffer and postfixed in 2% osmium tetroxide for 3 h at 4 °C. Then, the samples were dehydrated with a series of graded ethanol and embedded in epoxy resin. Ultrathin sections were obtained by ultramicrotome with diamond knives, stained with 2% uranyl acetate and lead citrate, and imaged with a JEOL 1400 Flash electron microscope. For image analysis, mitochondria were manually traced and quantified in the NIH ImageJ software (https://imagej.net/ij/index.html).

### Histological analysis

BAT and iWAT samples were fixed in 4% paraformaldehyde at 4 °C overnight, followed by embedding in paraffin and slicing into 3 μm sections. These sections were stained with H&E and visualized and photographed using a Keyence BZ-X710 microscope with the BZ-X Viewer software (Keyence) (https://www.keyence.com/landing/microscope/lp_fluorescence.jsp). Lipid droplet sizes within brown adipocytes were quantified using the NIH ImageJ software.

### Quantification of BAT triglyceride content

Triglycerides were extracted from BAT using a modified Folch's method (36). Briefly, BAT homogenization in cold saline was followed by extraction in 2:1 chloroform-methanol (v/v) solution. The extracted triglycerides were resuspended in 2-propanol and quantified using the LabAssay Triglyceride Kit (FUJIFILM Wako Pure Chemical Corporation), normalized to the protein content determined using a bicinchoninic acid protein assay kit (Thermo Fischer Scientific).

### Western blot analysis

Tissues were homogenized in radioimmunoprecipitation assay buffer supplemented with protease and phosphatase inhibitor cocktails (Sigma-Aldrich) using the gentleMACs Dissociator System (Miltenyi Biotec), followed by centrifugation at 17,000*g* for 15 min at 4 °C. The protein concentration was determined using a bicinchoninic acid protein assay kit (Thermo Fischer Scientific). The protein extracts were diluted in SDS sample buffer, denatured by heating (95 °C, 3 min), resolved on 4 to 20% gradient polyacrylamide gels (Cosmo Bio), and transferred onto nitrocellulose membranes (GE Healthcare). Membranes were blocked using Blocking One (Nacalai Tesque), followed by overnight incubation with specific primary antibodies at 4 °C. The primary antibodies used are listed in Table S2. Specific proteins were visualized on a film using horseradish peroxidase–conjugated anti-mouse IgG or anti-rabbit IgG secondary antibodies (Jackson ImmunoResearch), along with an enhanced chemiluminescence substrate kit (GE Healthcare). Band intensities were quantified using the NIH ImageJ software, with α-tubulin or HSP90 bands for loading normalization. For the detection of phosphorylated PKA substrate and HSL in BAT, β3 agonist CL316,243 (Sigma-Aldrich) was intraperitoneally injected into mice at 1 mg/kg body weight.

### RNA extraction and gene expression analysis (reverse transcription qPCR)

Total RNA was extracted from BAT and WAT using the TRIzol reagent and Direct-zol RNA Miniprep Plus Kit (Zymo Research) according to the manufacturer's instructions. One microgram of total RNA was used to synthesize complementary DNA (cDNA) using the High-Capacity cDNA Reverse-Transcription Kit (Applied Biosystems). Subsequently, cDNA levels were analyzed *via* quantitative polymerase chain reaction (qPCR) using the Power SYBR Green Master Mix in an ABI Step-One-Plus Real-Time PCR System (Applied Biosystems). Gene expression levels were quantified using the standard curve method (ΔΔCт) and normalized to mouse cyclophilin A expression. All primer sequences are listed in Table S3.

### Quantification of mtDNA copy number

mtDNA and nuclear DNA were purified from the BAT of 12-week-old mice using the QIAamp DNA Mini Kit (Qiagen), following the manufacturer's protocols. The DNA concentration and purity were measured using a NanoDrop spectrophotometer (Thermo Fischer Scientific). The DNA samples, diluted to 10 ng/μl with UltraPure water (Invitrogen), were analyzed by qPCR using a standard curve method to quantify the mtDNA abundance normalized to nuclear genomic DNA abundance. Specifically, mtDNA was quantified using primers targeting the NADH-ubiquinone oxidoreductase chain 1 gene (*mtND1*), while nuclear DNA was quantified using primers targeting the hexokinase 2 gene (*HK2*). The primer sequences for *mtND1* and *HK2* are listed in Table S3.

### BAT NE turnover

BAT NE turnover was determined by measuring the decline in tissue NE content after inhibiting catecholamine synthesis and subsequent reaccumulation through treatment with α-methyl-D, L-p-tyrosine (Santa Cruz Biotechnology, Inc) (37). Mice housed at 23 °C were injected with α-methyl-D, L-p-tyrosine (200 mg/kg, i.p.) at 10 AM before being exposed to cold at 5 °C. After 0 and 3 h of cold exposure, the mice were euthanized *via* cervical dislocation. BAT was rapidly removed, weighed, snap-frozen in liquid nitrogen, and stored at −80 °C. The NE content in the dissected BAT was determined using the Noradrenaline Research ELISA Kit (ImmuSmol) following the manufacturer's instructions.

### Determination of serum BCAA and blood glucose levels

Blood samples from tail veins were used to determine blood glucose levels using an automatic blood glucometer (Antsense Duo, Horiba). Serum was obtained by centrifuging the blood samples at 1200*g*, 4 °C for 10 min. The serum BCAA concentration was determined using the BCAA Assay Kit (Abcam) according to the manufacturer's protocol.

### RNA-seq and analysis

Interscapular BAT RNA was isolated from 10-week-old WT and *Txnip*^*−/−*^ mice acclimated to either 30 °C or 23 °C for 1 week, with triplicates for each group. RNA purity and integrity were confirmed using a bioanalyzer (Agilent Technologies). RNA-seq libraries were generated from 500 ng of total RNA using poly-A selection, and the NEBNext RNA Library Prep Kit (NEB), according to a standard Illumina protocol. The libraries were quantified using both an Agilent bioanalyzer and qPCR-based quantification (NEB). Sequencing was conducted with single-end 75-bp read length on an Illumina NextSeq 550 instrument, ensuring a depth of at least 20 million reads. The reads were aligned to the University of California Santa Cruz mm10 reference genome using STAR version 2.7.5a (38). Measuring gene expression involved using the “-count exons” command in Homer (39). Subsequent differential gene expression analysis was performed using DESeq2 version 1.8.2 (DESeq2 package; https://www.bioconductor.org/packages/release/bioc/html/DESeq2.html) (40). Genes exhibiting a fold change >1.25 or <0.8 and an adjusted *p* value < 0.05 were selected as the differentially expressed genes and visualized in a volcano plot. Gene Ontology analysis was performed using Metascape 3.5, and a heat map was constructed to visualize downregulated genes. Genome browser images were captured *via* IGV 2.16.0v, generating a representative track for each condition from the replicate tag data format files.

### ChIP-seq and analysis

BAT pads were minced in ice-cold PBS and dissociated into single-cell suspensions using the Adipose Tissue Dissociation Kit (Miltenyi Biotec). The resulting cell pellets were fixed first in disuccinimidyl glutarate for 30 min and then in 1% formaldehyde for 10 min at RT. Subsequently, cross-linking in cell pellets was quenched by adding 125 mM glycine for 5 min at RT, followed by washing twice with ice-cold 1 × PBS. The resulting cross-linked cell pellets were resuspended in ice-cold shearing buffer (50 mM Tris pH 8.0, 10 mM EDTA pH 8.0, 1% SDS, and protease inhibitor) and sonicated using a Biorupter (UCD300, Cosmo Bio) for six cycles of 30 s on and 30 s off. The sonicated chromatin lysates were diluted tenfold with dilution buffer (16.7 mM Tris pH 8.0, 1 mM EDTA pH 8.0, 167 mM NaCl, 1.1% Triton X-100, 0.01% SDS, and protease inhibitor). A portion was set aside for input DNA, while the remainder was incubated with the antibody against H3K27ac (CST#8173) at 4 °C overnight. The next day, the chromatin was immunoprecipitated with IgG paramagnetic beads (Invitrogen) at 4 °C with rotation for 6 h, and the collected beads were washed six times with cell wash buffer (50 mM Tris pH 7.5, 5 mM EDTA pH 7.5, 150 mM NaCl, and 0.5% NP-40), followed by two washes with cold 10 mM Tris [pH 8.0] and 1 mM EDTA buffer. Chromatin immune complexes were eluted twice in bead elution buffer (0.1 M NaHCO_3_ and 1% SDS) at RT with rotation for 15 min and then decrosslinked overnight in 0.2 M NaCl at 65 °C. The associated proteins were digested with proteinase K at 45 °C for 2 h, and the final DNA was purified using the MinElute PCR Purification Kit (Qiagen). ChIP-seq libraries were prepared using the NEBNext Ultra II DNA Library Prep Kit (NEB) and evaluated for their quality and quantity using a combination of Agilent bioanalyzer and DNA High Sensitivity chips (Agilent Technologies). Two biological ChIP replicates were pooled for sequencing on an Illumina NextSeq 550 system, employing a single-end 75-bp read length. The sequencing reads were aligned to the University of California Santa Cruz mm10 reference genome using Bowtie2 version 2.2.6 (https://bowtie-bio.sourceforge.net/bowtie2/index.shtml) (41). ChIP-seq peak calling and annotation were performed using the Homer (39) program. A motif enrichment analysis was conducted using the “findMotifsGenome” command in Homer within a 200-bp window with default options.

### Metabolomic analysis

The BAT pads were homogenized in acetonitrile/Milli-Q water containing internal standards (H3304-1002, Human Metabolome Technologies, Inc) using zirconia beads (5 and 3 mmϕ) and a Micro Smash bead shaker (TOMY SEIKO). After centrifugation at 2300*g*, 4 °C for 5 min, the upper aqueous layer of the homogenate was filtered through a millipore 5-kDa cut-off filter at 9100*g*, 4 °C for 120 min to remove macromolecules. The resulting filtrate was vacuum-dried and reconstituted in 50 μl of Milli-Q water for metabolome analysis.

Metabolome analysis was conducted using capillary electrophoresis time-of-flight (CE-TOF) mass spectrometry for cation analysis and CE-tandem mass spectrometry for anion analysis following established protocols (42, 43). Briefly, the CE-TOF-mass spectrometry and CE-tandem mass spectrometry analyses were performed using an Agilent CE system equipped with an Agilent 6210 TOF mass spectrometer (Agilent Technologies) and an Agilent 6460 Triple Quadrupole LC/MS (Agilent Technologies), respectively. Both systems were controlled by the Agilent G2201AA ChemStation software version B.03.01 for CE (Agilent Technologies; https://www.agilent.com/en/product/software-informatics), connected *via* a fused silica capillary (50 μm i.d. × 80 cm total length) with commercial electrophoresis buffers (H3301-1001 and I3302-1023 for cation and anion analyses, respectively; Human Metabolome Technologies Inc. [HMT]) serving as the electrolyte. The TOF mass spectrometer scanned from *m/z* 50 to 1000 (42), while the triple quadrupole mass spectrometer operated in the dynamic multiple reaction monitoring mode for compound detection. Peaks were extracted using MasterHands, an automatic integration software (Keio University) (44), and MassHunter Quantitative Analysis B.04.00 (Agilent Technologies; https://www.agilent.com/en/product/software-informatics/mass-spectrometry-software) to obtain peak information, including *m/z*, peak area, and migration time. Signal peaks were annotated according to HMT's metabolite database based on their *m/z* values and migration times. Metabolite concentrations were determined *via* normalization to internal standards and evaluated using standard curves with three-point calibrations for each standard compound. A principal component analysis was performed using HMT's proprietary and R programs (45). Detected metabolites were plotted on metabolic pathway maps using the VANTED software (https://cls.uni-konstanz.de/software/vanted/) (46).

### Statistical analyses

Data are presented as mean ± SD. GraphPad Prism 9 (https://www.graphpad.com/) was used for graphing and statistical analyses. We used unpaired two-tailed Student's *t* test for two-group comparisons and one-way or two-way ANOVA for multiple-group comparisons, followed by Bonferroni's *post hoc* test. Survival analysis was performed using the log-rank (Mantel–Cox) test. Further details of all statistical analyses are provided in the figure legends, and statistical significance was determined at *p* < 0.05.

## Data availability

All data required to evaluate the conclusions in the paper are presented in the paper and/or Supporting Information. RNA-seq and ChIP-seq data were deposited in the NCBI Gene Expression Omnibus under the accession number GSE251657. Requests for all data and materials should be submitted at ktanabe@yamaguchi-u.ac.jp.

## Supporting information

This article contains supporting information.

## Conflict of interest

The authors declare that they have no conflicts of interest with the contents of this article.
