# Supplementary material for: Txnip deficiency causes a susceptibility to acute cold stress with brown fat dysfunction in mice
Source: J Biol Chem. 2025 Feb 11;301(3):108293. doi: 10.1016/j.jbc.2025.108293 (PMC11938133; doi:10.1016/j.jbc.2025.108293)
Supplement: Supporting information [file mmc1.pdf]

Txnip deficiency causes a susceptibility to acute cold stress with brown fat dysfunction in mice

Meng Zou, Katsuya Tanabe, Kikuko Amo-Shiinoki, Daisuke Kohno, Syota Kagawa, Hideki Shirasawa, Kenji Ikeda, Akihiko Taguchi, Yasuharu Ohta, Shigeru Okuya, Tetsuya Yamada, Tadahiro Kitamura, Hiroshi Masutani and Yukio Tanizawa

Corresponding author: Katsuya Tanabe, MD, PhD
Email of the corresponding author: ktanabe@yamaguchi-u.ac.jp

This PDF file includes:

Figures S1 to S8
Tables S1 to S3

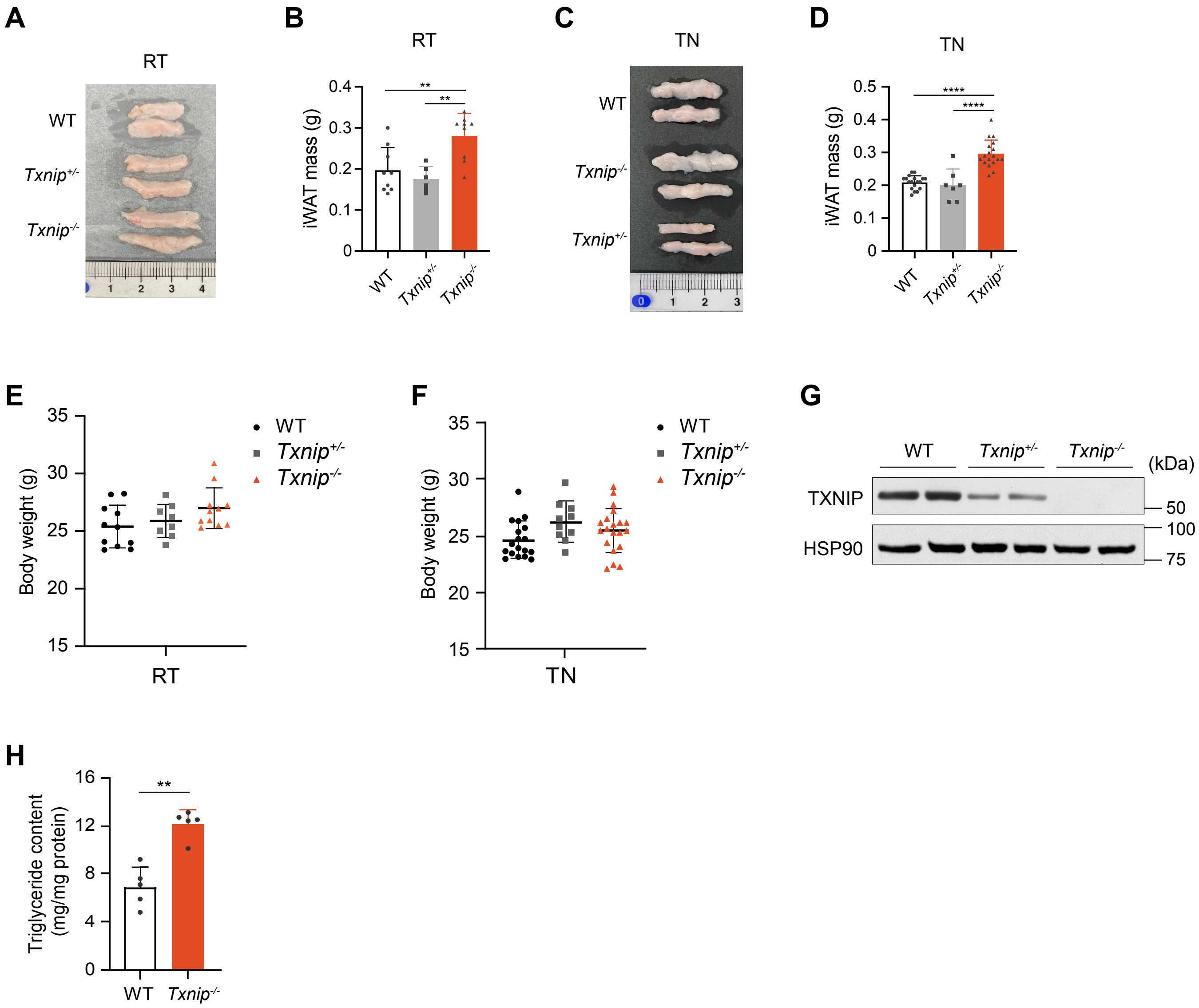

Figure S1. *Txnip*-deficient mice display increased inguinal WAT (iWAT) mass but no significant change in body weight on chow diet

A) Representative image of iWAT from 10-week-old wild-type (WT), *Txnip*-haploinsufficient (*Txnip*$^{+/-}$), and *Txnip*-deficient (*Txnip*$^{-/-}$) mice housed at room temperature (RT, 23°C); **B)** iWAT mass of 10-week-old WT, *Txnip*$^{+/-}$, and *Txnip*$^{-/-}$ mice housed at RT on a chow diet (n=9,6,9); **C)** Representative image of iWAT from 10-week-old WT, *Txnip*$^{-/-}$, and *Txnip*$^{+/-}$ mice acclimated to thermoneutral (TN, 30°C) conditions for 1 week; **D)** iWAT mass of 10-week-old WT, *Txnip*$^{+/-}$, and *Txnip*$^{-/-}$ mice acclimated to TN conditions on a chow diet (n=18,7,18); **E)** Body weight of 10-week-old WT, *Txnip*$^{+/-}$, and *Txnip*$^{-/-}$ mice housed at RT (n=11,8,11); **F)** Body weight of 10-week-old WT, *Txnip*$^{+/-}$, and *Txnip*$^{-/-}$ mice housed at TN for 1 week (n=18,10,20); **G)** Western blot of TXNIP in BAT from WT, *Txnip*$^{+/-}$ and *Txnip*$^{-/-}$ mice housed at RT. HSP90 was used as the loading control (n=2 per group); **H)** Quantification of triglyceride content in the BAT of WT and *Txnip*$^{-/-}$ mice acclimated to TN conditions for 1 week (n=5 per group). Data: Mean ± SD. Statistical analysis: one-way ANOVA with Bonferroni's *post hoc* test (**B, D, E, F**), unpaired Student's *t* test (**H**). *$p < 0.05$, **$p < 0.01$, ***$p < 0.001$, and ****$p < 0.0001$.

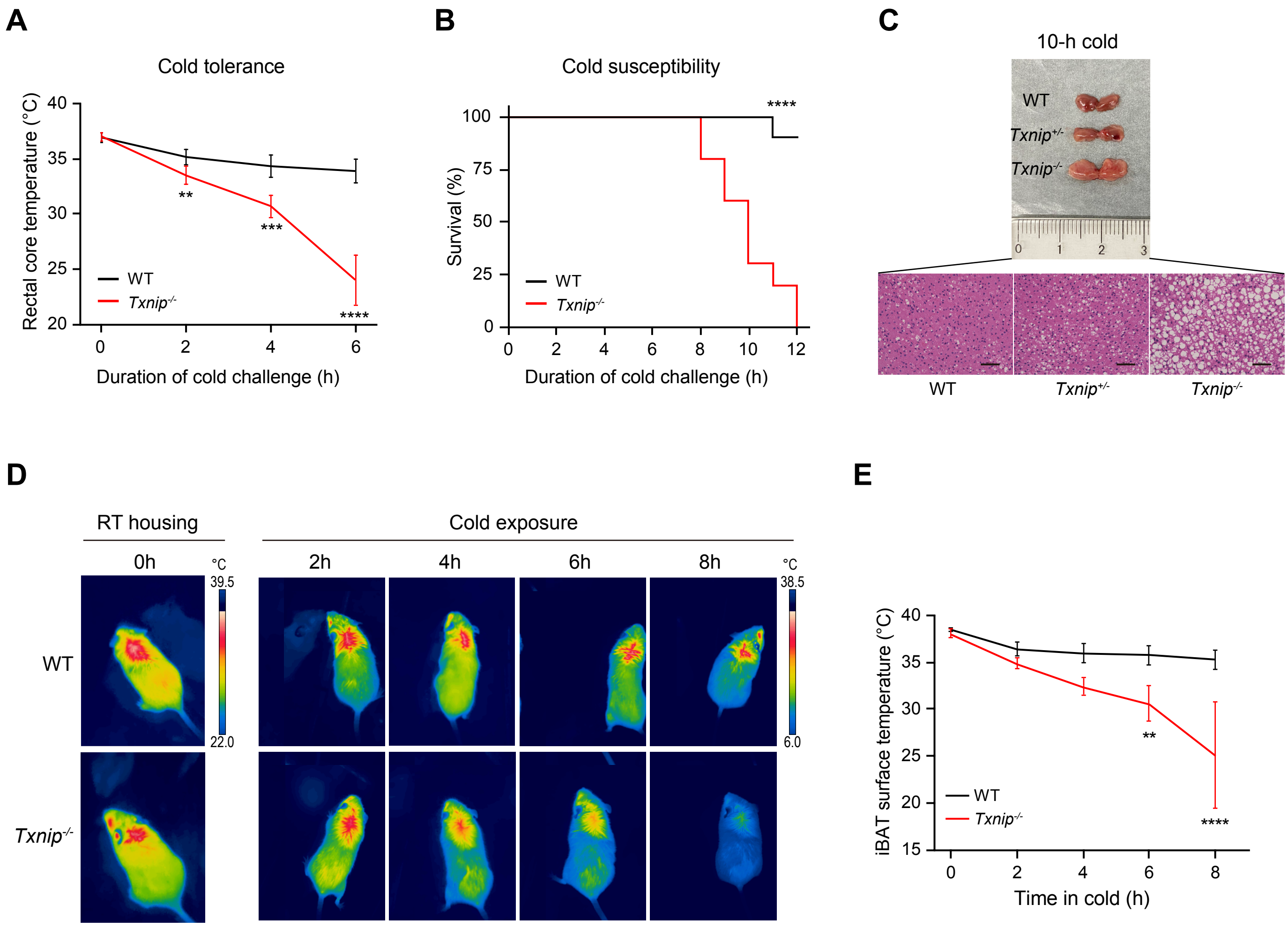

Figure S2. *Txnip*-deficient mice housed at RT show severe intolerance to acute cold exposure
A) Rectal core body temperature of WT and *Txnip*$^{-/-}$ mice exposed to acute cold at 5°C after pre-housing at RT (n=7 per group); **B)** Survival rate of WT and *Txnip*$^{-/-}$ mice exposed to acute cold at 5°C after pre-housing at RT (n=10 per group); **C)** Representative interscapular BAT image and H&E staining from WT, *Txnip*$^{+/-}$, and *Txnip*$^{-/-}$ mice exposed to acute cold for 10 h after pre-housing at RT. Scale bar represents 50μm; **D)** Representative infra-red images of WT and *Txnip*$^{-/-}$ mice pre-housed at RT, followed by acute cold exposure; **E)** Interscapular BAT surface temperatures of WT and *Txnip*$^{-/-}$ mice during acute cold exposure after pre-housing at RT (n=4 per group). Data: Mean ± SD. Statistical analysis: two-way ANOVA with Bonferroni's *post hoc* test (**A, E**), log-rank (Mantel-Cox) test (**B**). *p < 0.05, **p < 0.01, ***p < 0.001, and ****p < 0.0001.

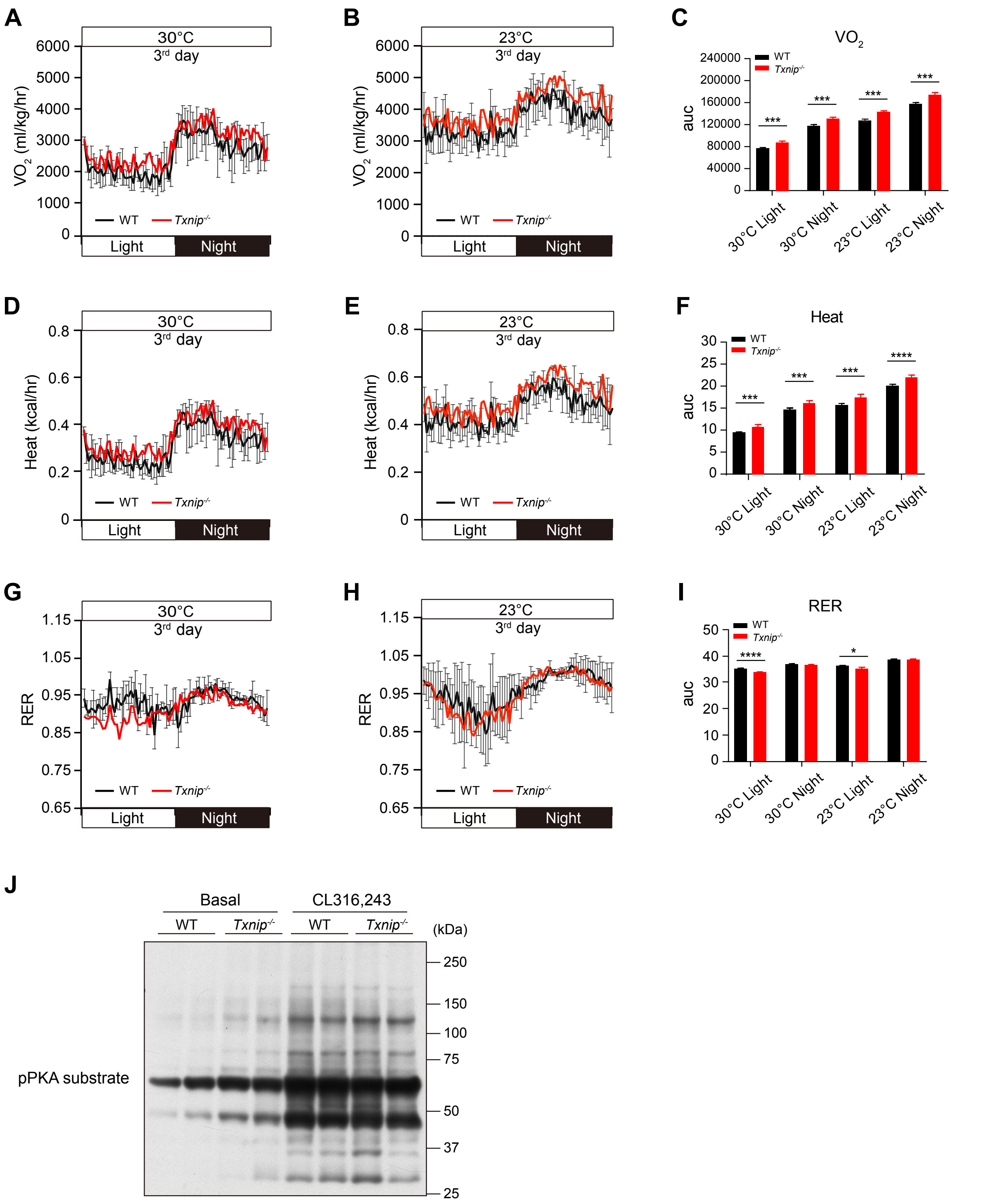

Figure S3. Whole-body energy expenditure in WT and *Txnip*$^{-/-}$ mice under TN and RT conditions
A and B) O_2 consumption rate (VO_2) in WT and *Txnip*$^{-/-}$ mice on the third day of TN housing **(A)** and RT housing **(B)** (n=4 per group); **C)** Total VO_2 in WT and *Txnip*$^{-/-}$ mice housed at TN and RT on the third day (n=4 per group); **D and E)** Heat production in WT and *Txnip*$^{-/-}$ mice on the third day of TN housing **(D)** and RT housing **(E)** (n=4 per group); **F)** Total heat production in WT and *Txnip*$^{-/-}$ mice housed at TN and RT on the third day (n=4 per group); **G and H)** Respiratory exchange ratio (RER) in WT and *Txnip*$^{-/-}$ mice on the third day of TN housing **(G)** and RT housing **(H)** (n=4 per group); **I)** Total RER in WT and *Txnip*$^{-/-}$ mice housed at TN and RT on the third day (n=4 per group); **J)** Western blot of phosphorylated PKA (pPKA) substrates in BAT lysates 5 min after injecting β3 agonist CL316,243 into WT and *Txnip*$^{-/-}$ mice housed at TN conditions.
Data: Mean ± SD. Statistical analysis: unpaired Student's *t* test (**A-I**). *$p < 0.05$, **$p < 0.01$, ***$p < 0.001$, and ****$p < 0.0001$.

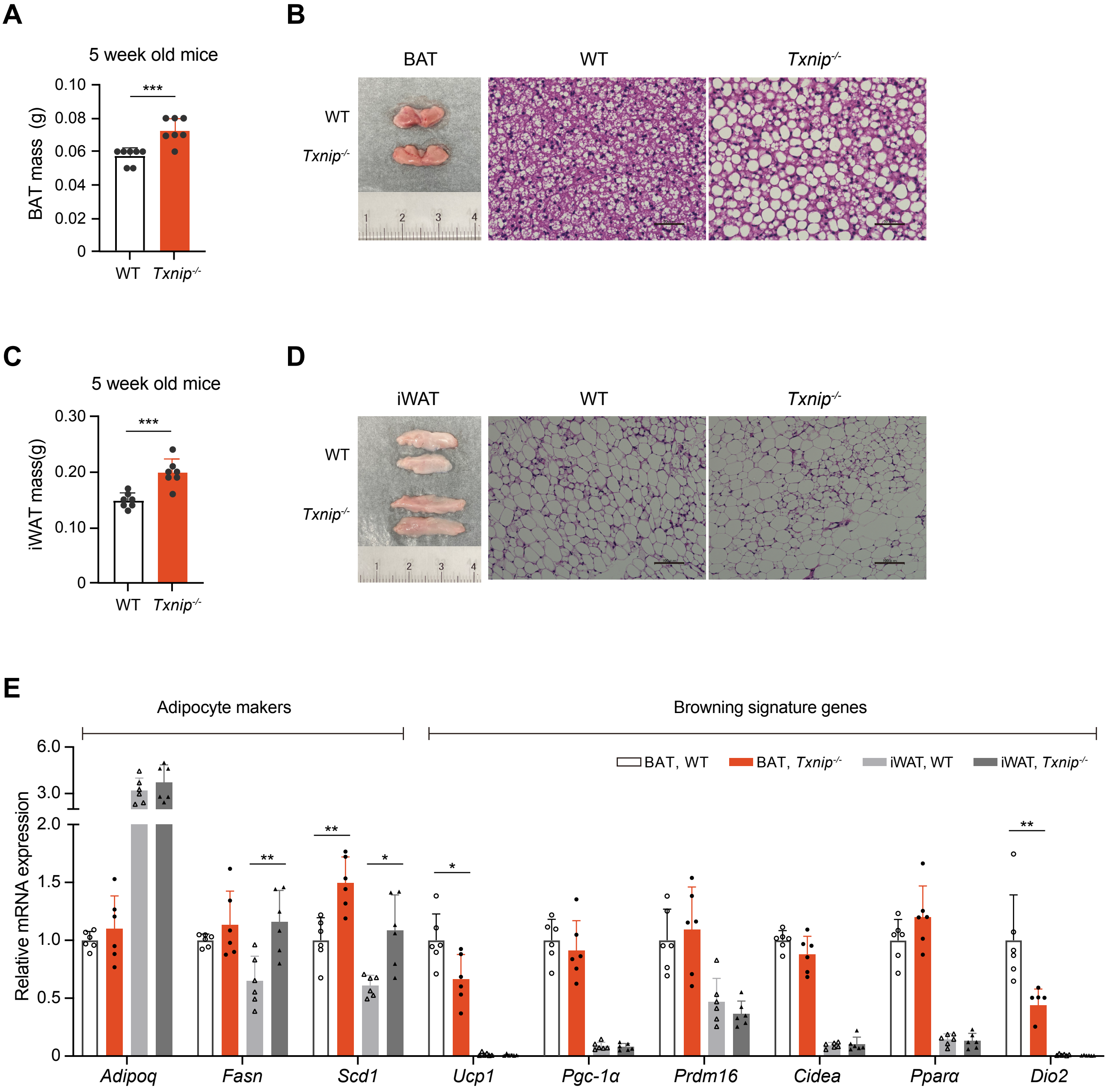

Figure S4. Adipose tissue morphology and browning signature gene expression in 5-week-old mice
A) Interscapular BAT mass of 5-week-old WT and *Txnip*$^{-/-}$ mice (n=7 per group); **B)** Representative macroscopic and H&E staining images of interscapular BAT from 5-week-old WT and *Txnip*$^{-/-}$ mice. Scale bar is 50μm; **C)** iWAT mass of 5-week-old WT and *Txnip*$^{-/-}$ mice (n=7 per group); **D)** Representative macroscopic and H&E staining images of iWAT from 5-week-old WT and *Txnip*$^{-/-}$ mice. Scale bar is 100μm; **E)** qPCR gene expression analysis of adipocyte markers and browning signature genes in interscapular BAT and inguinal WAT from 5-week-old WT and *Txnip*$^{-/-}$ mice (n=6 per group).
Data: Mean ± SD. Statistical analysis: unpaired Student's *t* test (**A, C, E**). *p < 0.05, **p < 0.01, ***p < 0.001, and ****p < 0.0001.

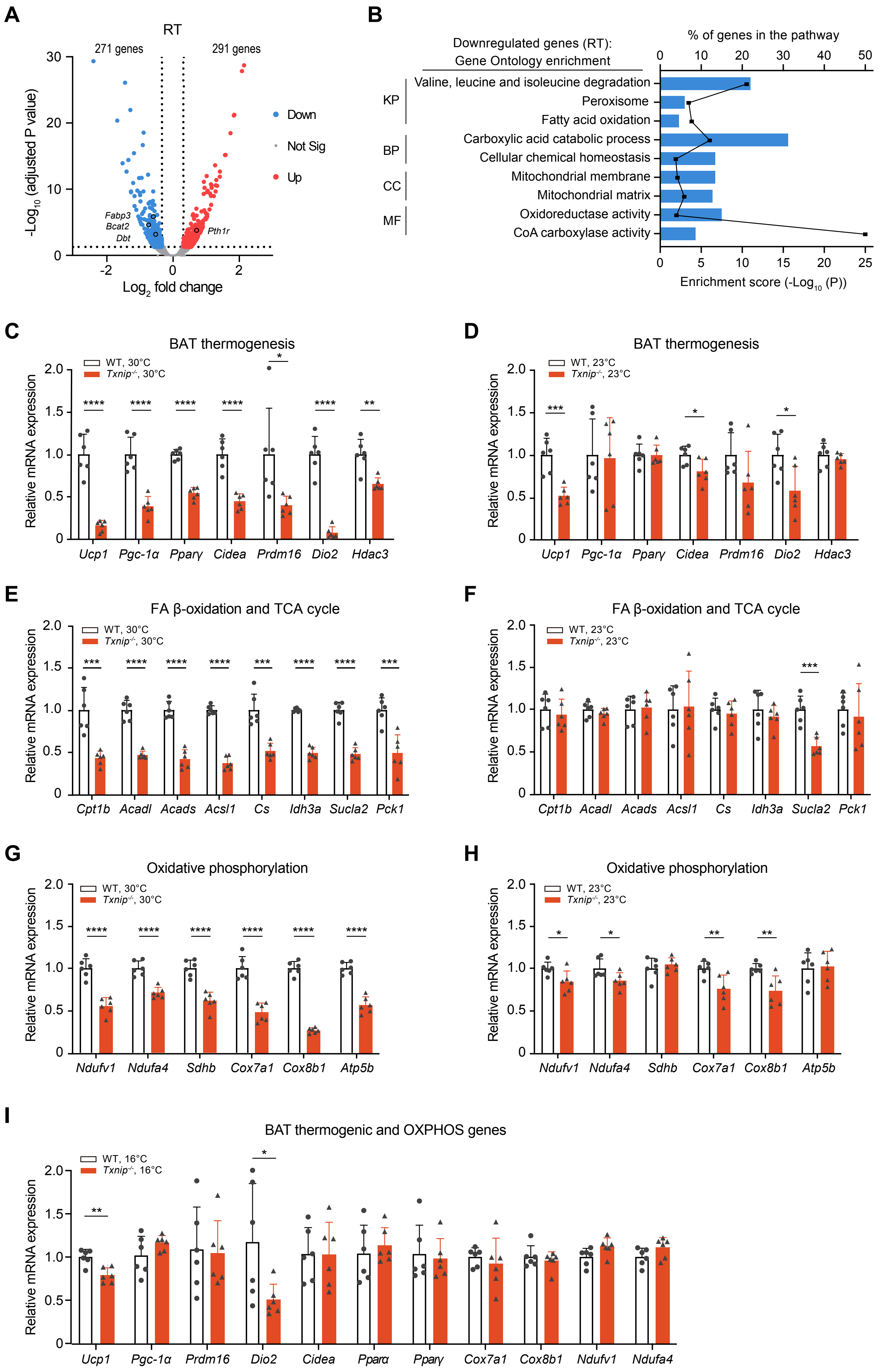
A
RT
271 genes
291 genes
-Log10 (adjusted P value)
Log2 fold change
Down
Not Sig
Up
Fabp3
Bcat2
Dbt
Pth1r
B
Downregulated genes (RT): Gene Ontology enrichment
% of genes in the pathway
KP
BP
CC
MF
Valine, leucine and isoleucine degradation
Peroxisome
Fatty acid oxidation
Carboxylic acid catabolic process
Cellular chemical homeostasis
Mitochondrial membrane
Mitochondrial matrix
Oxidoreductase activity
CoA carboxylase activity
Enrichment score (-Log10 (P))
C
BAT thermogenesis
WT, 30°C
Txnip-/-, 30°C
Relative mRNA expression
Ucp1
Pgc-1α
Pparγ
Cidea
Prdm16
Dio2
Hdac3
D
BAT thermogenesis
WT, 23°C
Txnip-/-, 23°C
Relative mRNA expression
Ucp1
Pgc-1α
Pparγ
Cidea
Prdm16
Dio2
Hdac3
E
FA β-oxidation and TCA cycle
WT, 30°C
Txnip-/-, 30°C
Relative mRNA expression
Cpt1b
Acadl
Acads
Acsl1
Cs
Idh3a
Sucla2
Pck1
F
FA β-oxidation and TCA cycle
WT, 23°C
Txnip-/-, 23°C
Relative mRNA expression
Cpt1b
Acadl
Acads
Acsl1
Cs
Idh3a
Sucla2
Pck1
G
Oxidative phosphorylation
WT, 30°C
Txnip-/-, 30°C
Relative mRNA expression
Ndufv1
Ndufa4
Sdhb
Cox7a1
Cox8b1
Atp5b
H
Oxidative phosphorylation
WT, 23°C
Txnip-/-, 23°C
Relative mRNA expression
Ndufv1
Ndufa4
Sdhb
Cox7a1
Cox8b1
Atp5b
I
BAT thermogenic and OXPHOS genes
WT, 16°C
Txnip-/-, 16°C
Relative mRNA expression
Ucp1
Pgc-1α
Prdm16
Dio2
Cidea
Pparα
Pparγ
Cox7a1
Cox8b1
Ndufv1
Ndufa4

Figure S5. *Txnip* is required for the expression of BAT thermogenesis and oxidative metabolism genes under basal conditions

A) Volcano plot of RNA-seq data showing *Txnip*-regulated BAT genes from *Txnip*$^{-/-}$ versus WT mice housed at RT (fold change >1.25 up (red) or fold change <0.8 down (blue)) (n=3 per group); **B)** Gene Ontology and pathway analysis of downregulated genes in *Txnip*$^{-/-}$ BAT under RT, identified by RNA-seq and selected by enrichment score. The bars represent the enrichment scores for down-regulated pathways. The lines represent the number of down-regulated genes as a percentage of the corresponding pathway. (KP, KEGG Pathway; BP, Biological Process; CC, Cellular Component; MF, Molecular Function); **C and D)** qPCR gene expression analysis of thermogenic genes in BAT from WT and *Txnip*$^{-/-}$ mice acclimated to TN for 1 week (**C**) or RT (**D**) (n=6 per group); **E and F)** qPCR gene expression analysis of FA β-oxidation and TCA cycle genes in BAT from WT and *Txnip*$^{-/-}$ mice acclimated to TN for 1 week (**E**) or RT (**F**) (n=6 per group); **G and H**) qPCR gene expression analysis of oxidative phosphorylation genes in BAT from WT and *Txnip*$^{-/-}$ mice acclimated to TN for 1 week (**G**) or RT (**H**) (n=6 per group); **I)** qPCR gene expression analysis of thermogenesis and oxidative phosphorylation genes in BAT from WT and *Txnip*$^{-/-}$ mice acclimated to 16°C for 1 week (n=6 per group).

Data: Mean ± SD. Statistical analysis: unpaired Student's *t* test (**C-I**). $^{*}p < 0.05$, $^{**}p < 0.01$, $^{***}p < 0.001$, and $^{****}p < 0.0001$.

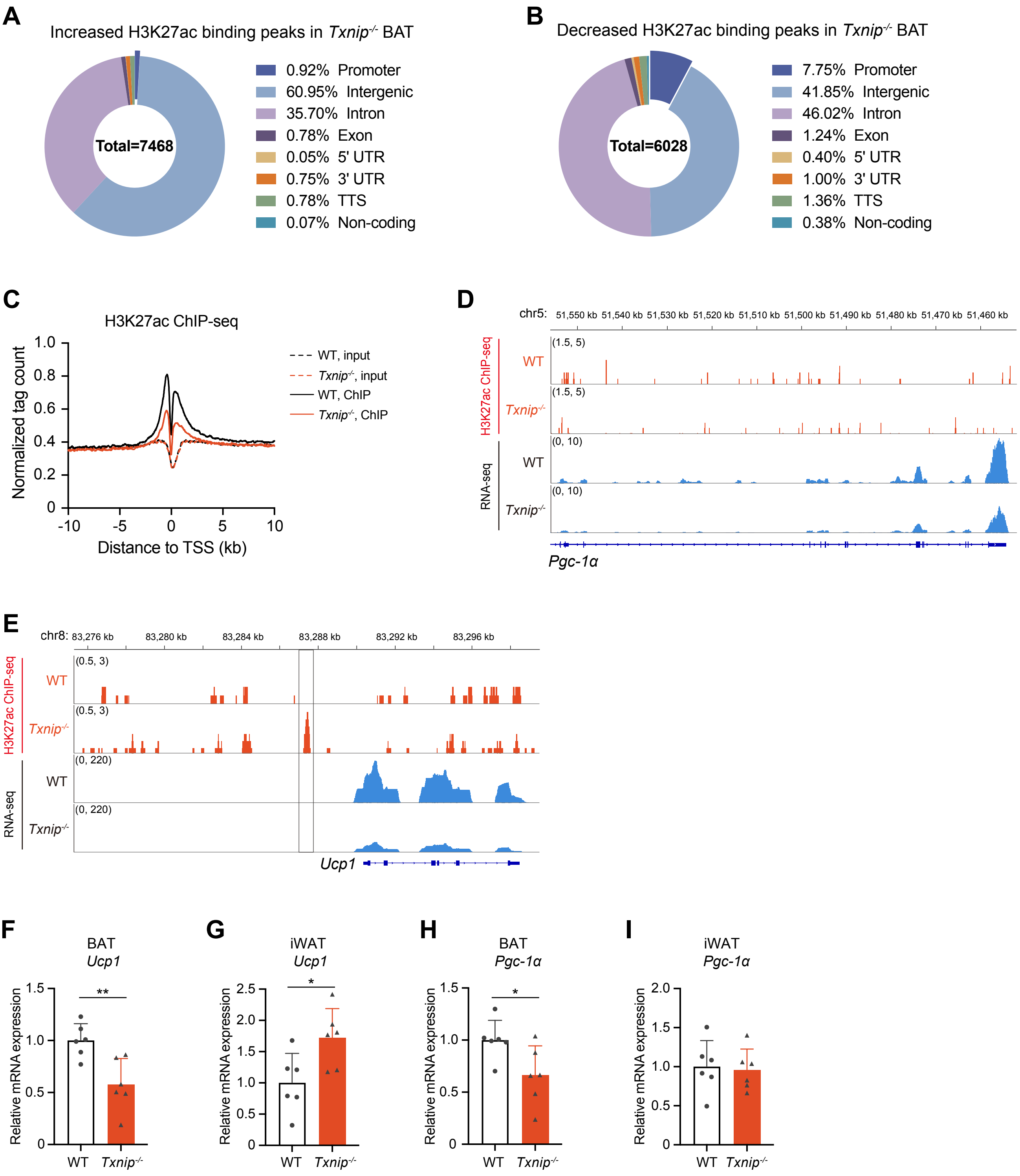

Figure S6. Transcriptional role of *Txnip* in BAT under basal conditions

A) Genomic distribution with increased H3K27ac binding peak in *Txnip*$^{-/-}$ versus WT BAT under TN conditions; **B)** Genomic distribution with decreased H3K27ac binding peak in *Txnip*$^{-/-}$ versus WT BAT under TN conditions; **C)** H3K27ac ChIP–seq profiles within ±10 kb of the transcription start sites of genes in BAT isolated from WT and *Txnip*$^{-/-}$ mice at TN conditions; **D and E)** Genome browser tracks displaying *Pgc1α* loci (**D**) and *Ucp1* loci (**E**) with ChIP-seq and RNA-seq data under TN conditions; **F and G)** *Ucp1* mRNA levels in BAT **(F)** and iWAT**(G)** from WT and *Txnip*$^{-/-}$ mice housed at TN conditions for 1 week, followed by 4-h acute cold exposure at 5°C (n=6 per group); **H and I)** *Pgc1α* mRNA levels in BAT **(H)** and iWAT**(I)** from WT and *Txnip*$^{-/-}$ mice housed at TN conditions for 1 week, followed by 4-h acute cold exposure at 5°C (n=6 per group).

Data: Mean ± SD. Statistical analysis: unpaired Student's *t* test (**F-I**). *p < 0.05, **p < 0.01, ***p < 0.001, and ****p < 0.0001.

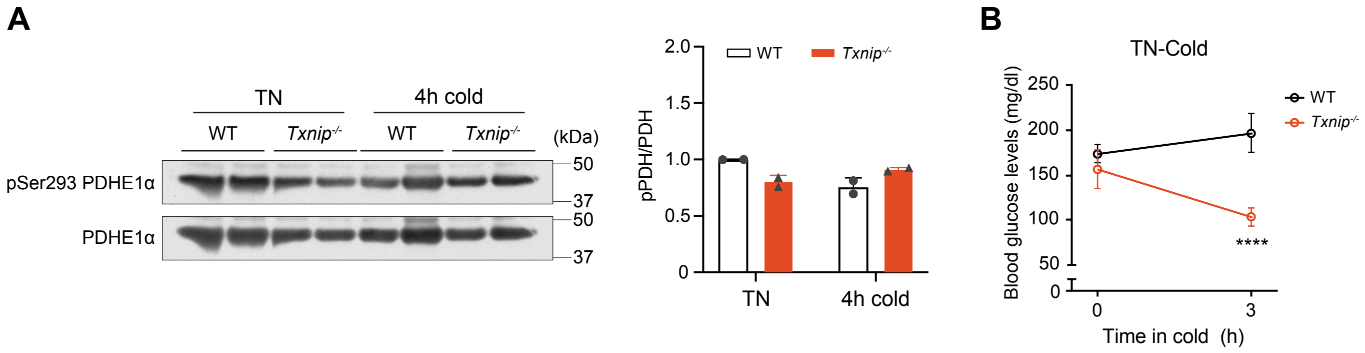

Figure S7. Glycolysis is maintained in *Txnip*-/- BAT upon acute cold exposure
A) Western blot and quantitation of phosphorylated pyruvate dehydrogenase at serine 293 (pSer293 PDHE1α) in BAT from WT and *Txnip*-/- mice housed at TN conditions for 1 week, followed by 4-h acute cold exposure at 5°C (n=2 per group); **B)** Changes in blood sugar levels in WT and *Txnip*-/- mice during acute cold exposure after TN acclimation (n=9 per group).
Data: Mean ± SD. Statistical analysis: unpaired Student's *t* test (**A**), two-way ANOVA with Bonferroni's *post hoc* test (**B**). *p < 0.05, **p < 0.01, ***p < 0.001, and ****p < 0.0001.

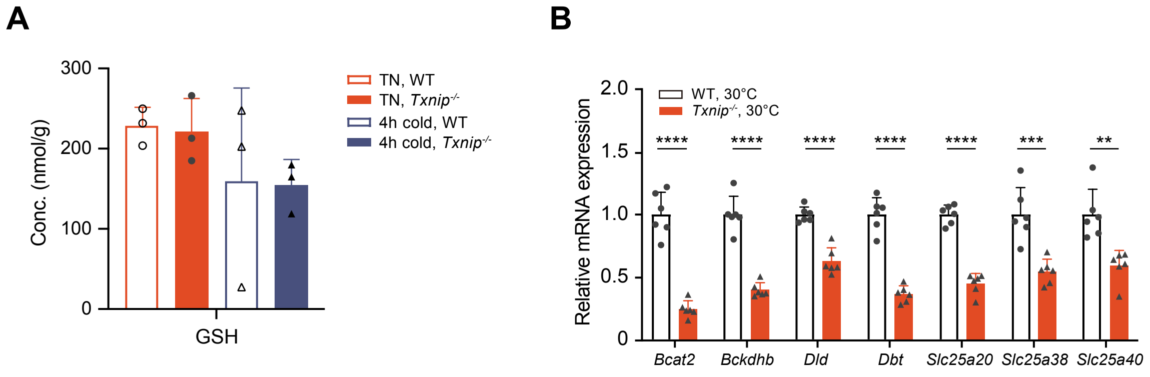

Figure S8. *Txnip* maintains basal expression of BCAA oxidation genes in BAT
A) Levels of GSH in BAT of WT and *Txnip*$^{-/-}$ mice housed at TN conditions for 1 week, followed by 4-h cold exposure at 5°C (n=3 per group); **B)** qPCR gene expression analysis of BCAA oxidation genes in BAT from WT and *Txnip*$^{-/-}$ mice housed at TN conditions for 1 week (n=6 per group).
Data: Mean ± SD. Statistical analysis: unpaired Student's *t* test. *p < 0.05, **p < 0.01, ***p < 0.001 and ****p < 0.0001.

Table S1. Main composition of the diets used in this study

	Standard chow		Modified Control diet		Modified ketogenic diet	
	g/100g	kcal%	g/100g	kcal%	g/100g	kcal%
Protein	23.1	25	11.9	12	17.9	12
Carbohydrate	55.3	62	73.4	77	9.4	6
Fat	5.1	13	4.4	10	56.7	82
Crude fiber	2.8	0	4.4	0	7.4	0
Moisture and crude ash	13.7	0	5.9	0	8.5	0
Total energy (kcal/100g)	359		381		619	

Table S2. List of antibodies used in this study

Protein	Dilution	Host, MW (kDa)	Antibody cat. no
TXNIP	1:1000	Rabbit mAb, 50	ab188865, Abcam
α-Tubulin	1:5000	Mouse mAb, 52	#3873, Cell Signaling Technology
p-HSL (Ser660)	1:1000	Rabbit pAb, 81, 83	#45804, Cell Signaling Technology
HSL	1:10,000	Rabbit mAb, 81, 83	#18381, Cell Signaling Technology
p-PKA substrate	1:1000	Rabbit mAb, IgG	#9624, Cell Signaling Technology
PPARγ	1:1000	Rabbit mAb, 53, 57	#2443, Cell Signaling Technology
PGC-1α	1:1000	Rabbit pAb, 100	AB3242, Sigma-Aldrich
UCP1	1:10,000	Rabbit mAb, 33	ab209483, Abcam
HSP90	1:1000	Rabbit pAb, 90	#4874, Cell Signaling Technology
p-PDHA1 (Ser293)	1:10,000	Rabbit pAb, 43	ab92696, Abcam
PDHE1a	1:1000	Rabbit mAb, 43	#3205, Cell Signaling Technology
H3K27ac	1:100	Rabbit mAb, 17	#8173, Cell Signaling Technology

p-, phosphorylated; pAb, polyclonal antibody; mAb, monoclonal antibody.

Table S3. List of primers used for real-time qPCR

Gene	Forward Primer 5'-3'	Reverse Primer 5'-3'
CyclophilinA	TGTGCCAGGGTGGTGACTTTAC	TGGGAACCGTTTGTGTTTGG
Ucp1	TCAGGATTGGCCTCTACGAC	TGCCACACCTCCAGTCATTA
Pgc-1α	CCCTGCCATTGTTAAGACC	TGCTGCTGTTCCTGTTTTC
Pparγ	GAACCTGCATCTCCACCTTATT	TGGAAGCCTGATGCTTTATCC
Prdm16	CAGCACGGTGAAGCCATTC	GCGTGCATCCGCTTGTG
Cidea	TGACATTCATGGGATTGCAGAC	GGCCAGTTGTGATGACTAAGAC
Pparα	ACAAGGCCTCAGGGTACCA	GCCGAAAGAAGCCCTTACAG
Dio2	CTGTGTCTGGAACAGCTT	CACTGGAATTGGGAGCAT
Adipoq	GCACTGGCAAGTTCTACTGCAA	GTAGGTGAAGAGAACGGCCTTGT
Fasn	TGGGTGTGGAAGTTCGTCAG	CTGTCGTGTCAGTAGCCGAG
Scd1	GCTCTACACCTGCCTCTTCG	GCCGTGCCTTGTAAGTTCTG
Hdac3	CCTGGAACAGGTGACATGTATGA	CGTAAGGGCACATTGAGACAATAG
Cpt1b	GATGCAGTTCCAGAGAATCC	CTTGTTCTTGCCAGAGCT
Acadl	GAAACCAGGAACTACGTGAAG	GCTGTCCACAAAAGCTCT
Acads	AAGTTTGGATCCGCACAGCAG	CAAGCTTTGGTGCCGTTGAG
Acsl	TGGGGTGGAAATCATCAGCC	CACAGCATTACACACTGTACAACGG
Cs	GGGACTTGTGTATGAGACTTCG	AGCCAAAATAAGCCCTCAGG
Idh3a	ACGGAAGGAGAATACAGTGG	GTACTCGAAGGCAAACTCTG
Sucla2	ACGGGTCGGACTCAGAAATG	ACATCCTTTGATCCTGGGCG
Pck1	TTGAACTGACAGACTCGCCCT	TGCCCATCCGAGTCATGA
Ndufv1	TCTCTGGCCATGTCAACCAC	CTGTGACACCACCAGCATGT
Ndufa4	AGCCATGGAACAAACTGGGT	ATTGTGCGGATGGCTTCTGA
Sdhb	GACGTCAGGAGCCAAAATGG	CTCGACAGGCCTGAAACTGC
Cox7a1	CAGCGTCATGGTCAGTCTGT	AGAAAACCGTGTGGCAGAGA
Cox8b1	GAACCATGAAGCCAACGACT	GCGAAGTTCACAGTGGTTCC
Atp5b	TTCAGGGGCACCAATCAAAATTC	CAACCTTTATCCCAGTCACCAGA
Bcat2	TTCCAGAACCTCACGCTACAC	TAGCAGAACGTAGCATCCTGTC
Bckdhb	GCCAAAGACCCCACTGCAGTAA	GGATTTCCGCAATAGCTGTAGCACC
Dld	GCAGATCAACCAATTGAGGCTGACG	GCATGTTCCACCTAGTGTCTC
Dbt	CCAACATTGGATCAATCGGTGG	CTGTGATCCGCTGACCAGCTCAC
Slc25a20	GTGCTCACACTCATGCGAGA	CGCTGAGGTCACTGACACTC
Slc25a38	CGTCCCCAGTGATCGAGAAG	GGAAGAGGAGCGTGGAACAG
Slc25a40	TTCACCTCGTGGTCGACATC	GAGTGCTTGCCTAACATGCC
mtND1	GTGGCTCATCTACTCCACTGA	TCGAGCGATCCATAACAATAA
HK2	CCAGGGCTGTAGGAACATGG	ACAGATAGCAAGCAGCCTCG
